# Sex Specificity in the Mixed Effects of Blood Heavy Metals and Cognitive Function on Elderly: Evidence from NHANES

**DOI:** 10.3390/nu15132874

**Published:** 2023-06-25

**Authors:** Shuaixing Song, Nan Liu, Guoxu Wang, Yulin Wang, Xiaoan Zhang, Xin Zhao, Hui Chang, Zengli Yu, Xiaozhuan Liu

**Affiliations:** 1 Center for Clinical Single-Cell Biomedicine, Henan Provincial People’s Hospital, People’s Hospital of Zhengzhou University, Zhengzhou 450003, China; 2 College of Public Health, Zhengzhou University, Zhengzhou 450001, China; 3 Institute of Environment and Health, South China Hospital of Shenzhen University, Shenzhen 518000, China; 4 The Third Affiliated Hospital of Zhengzhou University, Zhengzhou 450052, China

**Keywords:** heavy metals, mixture, cognitive function, joint effect, sex-specific

## Abstract

The way that males and females react to environmental exposures and negative impacts on their neurological systems is often different. Although previous research has examined the cognitively impairing effects of solitary metal exposures, the relationship between metal mixtures and cognitive function, particularly when considering an individual’s sex, remains elusive. This study aimed to investigate the sex differences in the association between multiple metal combinations and cognitive function in older Americans. This research employed the 2011–2014 NHANES survey of elderly Americans. The association between five mixed metals and four cognitive tests (the animal fluency test (AFT), the digit symbol substitution test (DSST), the instant recall test (IRT), and the delayed recall test (DRT)) were investigated with generalized linear regression model (GLM), Bayesian kernel machine regression model (BKMR), weighted quantile sum regression model (WQS), and quantile g-computation regression model (Qgcomp). A total of 1833 people, including 883 males and 950 females, enrolled in this cross-sectional study. We discovered that blood lead and blood cadmium were negatively associated with cognitive performance, while blood selenium demonstrated a positive association with cognitive function in older people. The negative relationship of heavy metal combinations on cognitive function might be somewhat reduced or even reversed via selenium. The IRT, AFT, and DSST are three of the four cognitive tests where men had more dramatic positive or negative results. There was a sex-specific connection between blood metal ratios and cognitive function among older Americans, as evidenced by the more significant relationship between mixed metals and cognitive performance in men (either positively or negatively). These results emphasize the impacts of ambient heavy metal exposure on cognitive function by employing sex-specific methods.

## 1. Introduction

Older people frequently experience cognitive impairment, which harms their quality of life and social interactions. Declining cognitive function significantly increases both personal and public health burdens as the percentage of the elderly population rises quickly [1]. According to reports, several physical, psychological, social, and living variables and health factors combine with inherited and external factors to trigger cognitive decline [2]. Current evidence indicates that environmental factors, such as heavy metals, may impact the cognitive function of older people [3,4]. Although metals are naturally existing elements, diffuse air pollution, cigarette smoke, and contaminated food and water are the main artificial sources of exposure [5]. Individuals with elevated levels of heavy metals face a risk of developing renal injury, neuropathy, coronary disease, and other serious illnesses [6].

When examining illness burden, gender differences in metal toxicity should be taken into account. Metals can cause cellular reactive stress after entering the body through many different paths, leading to various physiological, metabolic, and behavioral dysfunctions. Differences in the dopaminergic system and neuroimmune axis make males more vulnerable to exposure to metals such as mercury [7]. Evidence for sex-specific neurotoxic effects of manganese may also derive in part from the metal’s differential assimilation, absorption, and storage [8]. It has been documented that exposure to dangerous metals, including lead, cadmium, tungsten, and manganese, is related to lowered cognitive function [7,9,10,11]. However, it has also been discovered that some heavy metals, such as selenium and zinc, can benefit cognitive function [12,13]. Laboratory data suggest that there might be sex disparities regarding how heavy metals affect cognitive performance due to variations in hormone levels, body makeup, and brain architecture [14,15]. Given that there are sex disparities in both the prevalence and manifestations of cognitive-related diseases, as well as in how well these disorders respond to treatment, it is crucial to examine how sex differences in changes in cognitive ability relate to these differences. The sex-specific relationship between mental exposure and cognitive function [16,17], however, is still poorly understood since there lacks sufficient population-based research that concentrates on such. The findings of this study regarding sex variations in cognitive function may be crucial for understanding cognitive disease, but this epidemiological study has no individual predictive value.

Humans are exposed to multiple environmental chemicals concurrently, which can contribute to interactions between co-managed chemicals and confound the study of effects. Individuals are inevitably exposed to multiple metals at once, and these metals can interact with one another. The majority of previous epidemiological research has been on the relationship between individual metal exposures and cognitive function. Only single pollutant models have been used in studies on the effects of heavy metal exposure on cognitive function; this may have an impact on effect estimates by neglecting mixed data [9,18]. Combinations of metals might function either effectively or antagonistically, as different metals may facilitate or prevent the absorption of other metals [3,4]. According to the research conducted on teenagers in Bangladesh, selenium is favorably correlated with cognition, whereas manganese, arsenic, and cadmium are negatively correlated with working memory, visual recognition, and memory [19]. Prenatal metal combination exposure has been linked to neurocognitive development in children, according to studies on neonates [20,21,22]. In this study, we investigated the correlation between cognitive function and five heavy blood metals in senior people participating in the National Health and Nutrition Examination Survey (NHANES) between 2011 and 2014. Additionally, we looked into correlations between sex and cognitive function, pinpointing individual metals with the most important mixtures. We expect that the academic and clinical communities will benefit greatly from the information this study will contribute, which should result in more efficient prevention for both men and women.

## 2. Materials and Methods

### 2.1. Study Design and Population

**Figure 1:**
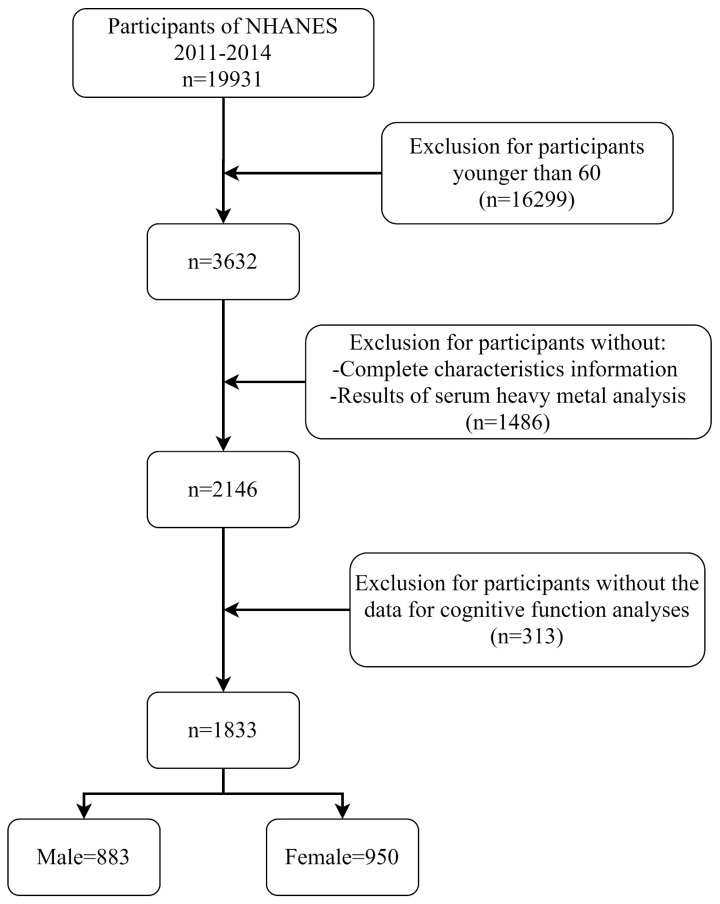
The specific selection process for inclusion in the study.

The National Health and Nutrition Examination Survey (NHANES) is a cross-sectional population health survey conducted by the National Center for Health Statistics (NCHS) of the Centers for Disease Control and Prevention (CDC) among the non-institutionalized U.S. population [23]. The representative samples for this investigation were selected through a complex multi-stage, hierarchical sampling approach. The protocol for NHANES has been approved by a review committee affiliated with CDC. All participants completed informed consent forms. This study combined and evaluated demographic, examination, laboratory, and questionnaire data from participants enrolled in the NHANES. Our study was limited to 3632 participants aged 60 or older out of 19,931 respondents who participated in the NHANES between 2011 and 2014. Throughout the investigation, we removed respondents with missing sociological characteristics and laboratory test results for serum heavy metals (n = 1486), as well as older persons who lacked four complete cognitive assessments (n = 313). Finally, this study comprised a total of 1833 individuals aged 60 or older, including 883 males and 950 women. The specific selection process for inclusion in the study is shown in Figure 1.

### 2.2. Measurements of Blood Heavy Metals

The processing, storage, and shipment of whole blood specimens to the NCHS, CDC for study. Serum concentrations of lead (Pb), cadmium (Cd), mercury (Hg), manganese (Mn), and selenium (Se) were measured employing inductively coupled plasma mass spectrometry with quadrupole ICPu20MS technology. The serum heavy metal concentrations of the participants in this investigation are provided in Table 1. In order to comply with the standards of statistical analysis, the serum heavy metal assay values were log-transformed during subsequent data processing according to the results of the statistical description.

### 2.3. Measurement of Cognitive Performance

The animal fluency test (AFT), the digit symbol substitution test (DSST), and the Consortium to Establish a Registry for Alzheimer’s Disease (CERAD) word list learning test were employed to evaluate the cognitive function of participants. The reliability of these tests in determining the cognitive level of the individuals is quite considerable [24]. The instant recall test (IRT) and delayed recall test (DRT) are part of the CERAD word learning tests utilized to evaluate the immediate and delayed acquisition of new linguistic material. AFT was used to measure executive function, wherein participants had 60 s to identify as many animals as they could, with the total number of adequately identified animals counting toward the final score. Finally, the researchers employed the DSST to evaluate reaction speed, sustained attention, and working memory among participants, with the total number of adequately matched numbers and symbols serving as the score. There is currently no golden standard for determining poor cognitive performance on the four previous cognitive tests, with higher scores on all tests indicating superior cognitive performance.

### 2.4. Covariates

In addition to the five serum heavy metals described above, we investigated a number of potentially confounding variables, including age (60–69 years; 70–79 years; 80+ years), sex (male; female), race/ethnicity (non-Hispanic White; non-Hispanic Black; other Hispanic; other/Multi-racial; Mexican; Other Race, Including Multi-Racial), education level (less than 9th grade; 9–11th grade; high school grad/GED; some college or AA degree; college graduate or above), smoking status (never smoker; current smoker; former smoker), alcohol intake (1–5 drinks/month; 5–10 drinks/month; 10+ drinks/month; non-drinker).

### 2.5. Statistical Analysis

The data analysis in this study was performed with R (version 4.2.2, R Core Team, Vienna, Austria). Categorical variables are expressed as the number of instances (n) and frequency (%), whereas non-normally distributed continuous variables are expressed as the median (IQR = Q_75_ − Q_25_). For group comparisons of data with a normal distribution, we utilize Student’s *t*-test, or for group comparisons of skewed variables, we used the Wilcoxon rank sum test. The chi-square test was performed to examine the variations in rates for categorical variables amongst groups. *p* < 0.05 for a two-sided test was considered statistically significant. In this investigation, a subgroup analysis was conducted to investigate the sex differences in the relationship between serum heavy metals and cognitive function in older persons, taking into account the substantial variations between the four cognitive tests in the sex stratification. In addition, Pearson correlation analysis was employed to examine relationships between blood heavy metals.

#### 2.5.1. Statistical Model 1: Generalized Linear Regression Model (GLM)

During the initial stage, this research employed weighted generalized linear regression models in a complex sampling environment to investigate the connection between four cognitive scores and serum heavy metal concentrations separately, adjusting for relevant demographic and behavioral confounders. Serum heavy metals were included in the analysis of generalized linear regression models with continuous and categorical variables due to the likelihood of nonlinear correlation between serum heavy metals and outcome. Dominance ratios and related 95% confidence intervals summarize the statistical results. Model was adjusted for age, sex, race/ethnicity, education level, smoking status, and alcohol intake.

#### 2.5.2. Statistical Model 2: Bayesian Kernel Machine Regression (BKMR) Model

The combined effects and potential interactions between serum heavy metals and cognitive function were then examined via BKMR statistical modeling [22,25]. The nonlinear relationship between exposure and outcome was investigated in this research using exposure–response cross-sections for a single variable and outcome while other variables were held constant at the median. Bivariate exposure–response profiles represent how mixture compositions interact, which could be understood as potential interactions between the slope of the curve for one chemical at the 10th, 50th, and 90th modifications of another chemical (with the remaining variables fixed at the median). The association plot of the overall effect of the mixture with outcome shows the change in estimated outcome when all exposure variables are set at different percentiles simultaneously compared to when they are fixed at the median. Using the Markov Chain Monte Carlo method, iteration was set at 30,000.

#### 2.5.3. Statistical Model 3: Weighted Quantile Sum (WQS) Regression Model

The cumulative impact of metal mixture components on cognitive function was estimated by employing WQS regression. The WQS statistical model for multiple regression in high-dimensional data sets calculates the effects of all exposure factors on outcomes by constructing a weighted index and determining whether that index is related to outcomes [26]. The relative intensity of the weights given to each variable by the model allows the researcher to subsequently evaluate the contribution of each environmental chemical to the overall index impact, allowing for the identification of significant substances in the mixture.

#### 2.5.4. Statistical Model 4: Quantile g-Computation (Qgcomp) Regression Model

We further employed the Qgcomp model to overcome the limitations of the WQS regression model on the direction of association. The G-computation procedure has some advantages relative to traditional regression, including the decoupling of confounding adjustment and effect estimation and the causal parameter interpretation [27]. Qgcomp combines the inferential simplicity of weighted quantile sum regression with the flexibility of g-computation without the requirement of homogeneity assumption and the linearity and additivity of exposure. Qgcomp is a straightforward and computationally efficient method for estimating the association between a combination of exposures and the desired health outcome. Qgcomp can be used to consistently estimate effects of the exposure mixture in settings in which WQS regression may be biased or inconsistent but also yield equivalent estimates with WQS regression in large samples when its assumptions hold [28]. Using the qgcomp.noboot function, a linear model of cognitive function was fitted to evaluate the total effect through allocating positive or negative weighting indices to each blood heavy metal by segmenting each metal into quartiles. In order to determine the mixing effect’s linearity and display it using a g computation to show the mixed effect, the qgcomp.boot function (R package, “qgcomp”) was used.

## 3. Results

### 3.1. Characteristics of the Study Participants

This study enrolled 1833 eligible participants, including 883 men and 950 women. As shown in Table 2, the results of the sex subgroup analysis differed in terms of age, education, smoking, and alcohol consumption. Additionally, males were far more inclined to smoke and consume alcohol than women. There were significant sex differences on three of the four cognitive assessments, with males achieving lower cognitive scores than females (IRT, DRT, DSST). All five serum heavy metal concentrations exhibited a skewed distribution, as presented in Table 1 of the statistical description of serum heavy metal concentrations. Analysis of correlation revealed no correlation between serum heavy metal concentrations included in the study (Figures S1–S3).

### 3.2. Single Metal Exposures and Cognitive Function

According to the results provided by Tables S1–S6, a correlation existed between blood selenium and cognition. Blood selenium at Q2 equates was found to have the most positive effect on cognitive function according to the analysis of GLM based on quartiles of exposure variables. Additionally, blood lead was negatively correlated with DSST scores across all variables ((β(95% CI): −0.52 (−0.93, −0.11), *p* < 0.01); blood cadmium was negatively associated with IRT ((β(95% CI): −0.73 (−1.50, −0.03), *p* = 0.03); blood cadmium was negatively correlated with DSST (β(95% CI): −3.00 (−5.10, −0.99), *p* = 0.03); blood manganese was negatively correlated with AFT ((β(95% CI): −0.05 (−0.11, −0.01), *p* = 0.04).

### 3.3. Multi-Metal Exposures and Cognitive Function

#### 3.3.1. Multi-Metal Exposures and Cognitive Function: BKMR Model

**Figure 2:**
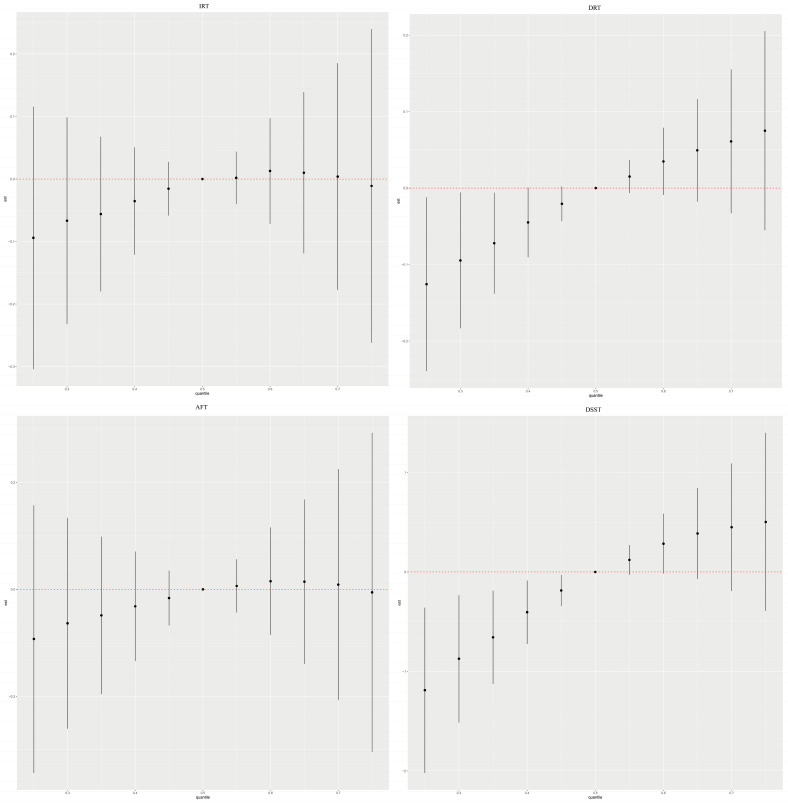
Combined effects of the metal as a mixture on cognitive function in elderly people.

**Figure 3:**
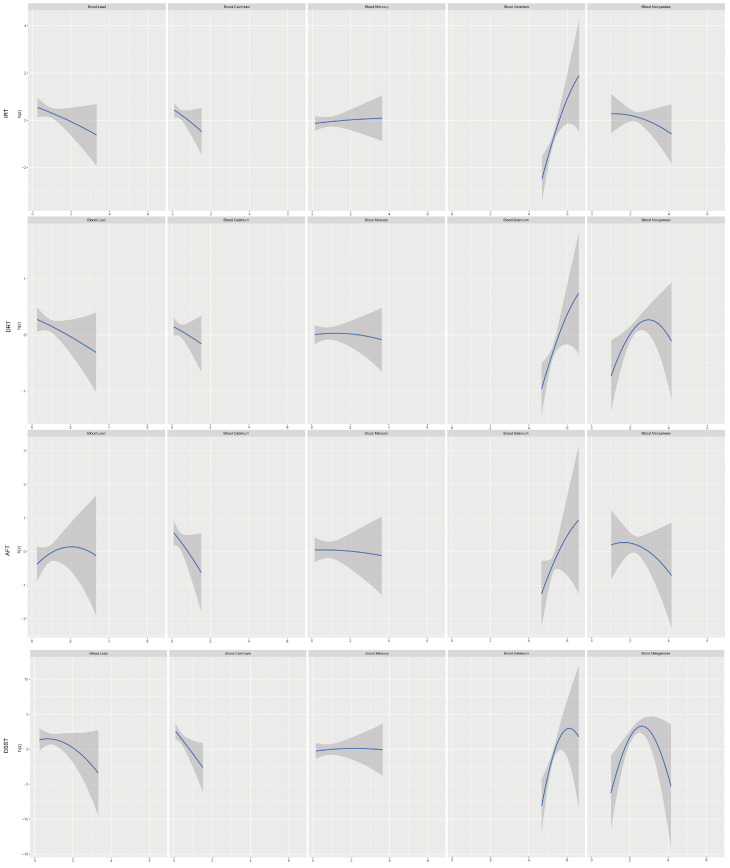
Univariate exposure–response functions and 95% confidence interval for each heavy metal with the other metals fixed at the median in elderly people.

We examined the relationship between five blood heavy metal co-exposures and cognitive function through the BKMR model (Figure 2, Figure 3, Figures S4 and S5). The results of four cognitive assessments were positively correlated with blood heavy metal co-exposure among the male participants. The general impact of blood heavy metal in the female subset was positively correlated with DRT and DSST scores but negatively correlated with AFT. Other blood heavy metals were set at their 50th percentile exposure amounts to evaluate unilateral impacts in univariate exposure–response functions (Figures S6 and S7; Table 3 and Tables S7–S9). Blood selenium was found to be a significant component for improved cognitive performance in the study, and its association with cognitive performance was more remarkable in males (IRT: 0.99; DRT: 0.71; AFT: 0.81; DSST: 0.99) than in women (IRT: 0.56; DRT: 0.63; AFT: 0.25; DSST: 0.71). Additionally, there was a difference between male and female groups in the negative association between blood lead, blood cadmium, and blood manganese and cognitive capacity. For instance, the DRT scores of blood manganese among males showed an “inverted U-shaped” curve; however, the relationship with blood manganese in females was DSST. The DSST scores of blood lead and females exhibited an “inverted U-shaped” curve, yet the connection was falling in males. Notably, when the other five metals were set at the 10th, 50th, and 90th percentiles, blood manganese and blood selenium may have possible associations with other metal concentrations (Figures S8–S10).

#### 3.3.2. Multi-Metal Exposures and Cognitive Function: WQS Model

**Figure 4:**
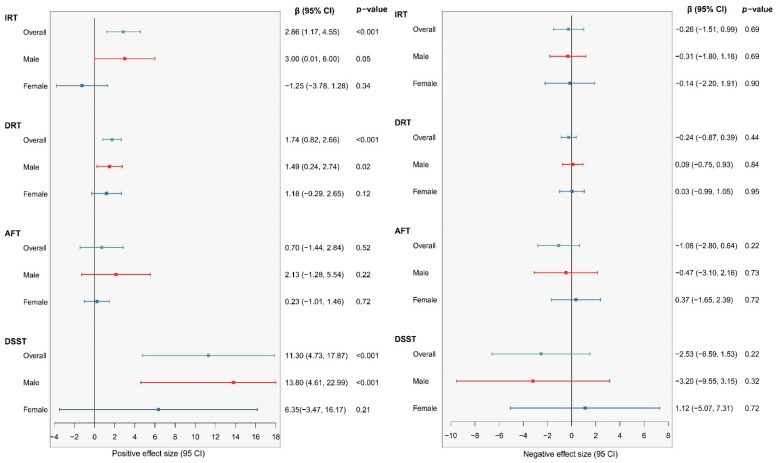
Associations between blood heavy metals and cognitive function by WQS regression model in NHANES 2011–2014. Cyan: whole population; Red: male; Blue: female.

**Figure 5:**
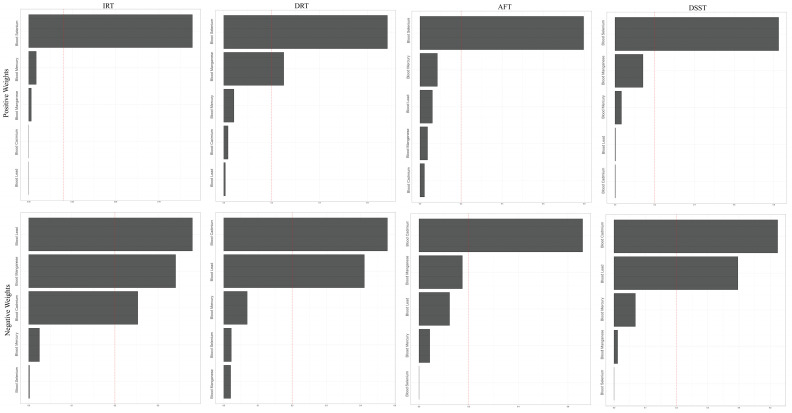
WQS model regression index weights for blood heavy metals and cognitive function in elderly people. Models were adjusted by age, race/ethnicity, education level, alcohol intake, and smoking status.

We initially examined the combined effect of serum heavy metals on cognitive function. As observed in Figure 4, the IRT, AFT, and DSST are three of the four cognitive tests where men had more dramatic positive or negative results. Likewise, the beneficial effects of five blood heavy metals on cognitive performance in males were further supported by favorable WQS model analysis results (IRT: 3.00 (0.01, 6.00); DRT: 1.49 (0.24, 2.74); DSST: 13.80 (4.61, 22.99). No statistically meaningful variations were found in the negative WQS model. According to the weighing study of all the demographic factors, blood selenium had the most significant protective impact on brain performance, while blood cadmium and blood lead had the opposite effects (Figure 5, Table 4). Similar tendencies were observed in the sex subgroups. (Figures S11 and S12, Tables S10–S12). Remarkably, blood lead produced a protective effect in the female AFT test results but not blood selenium.

#### 3.3.3. Multi-Metal Exposures and Cognitive Function: Qgcomp Model

**Figure 6:**
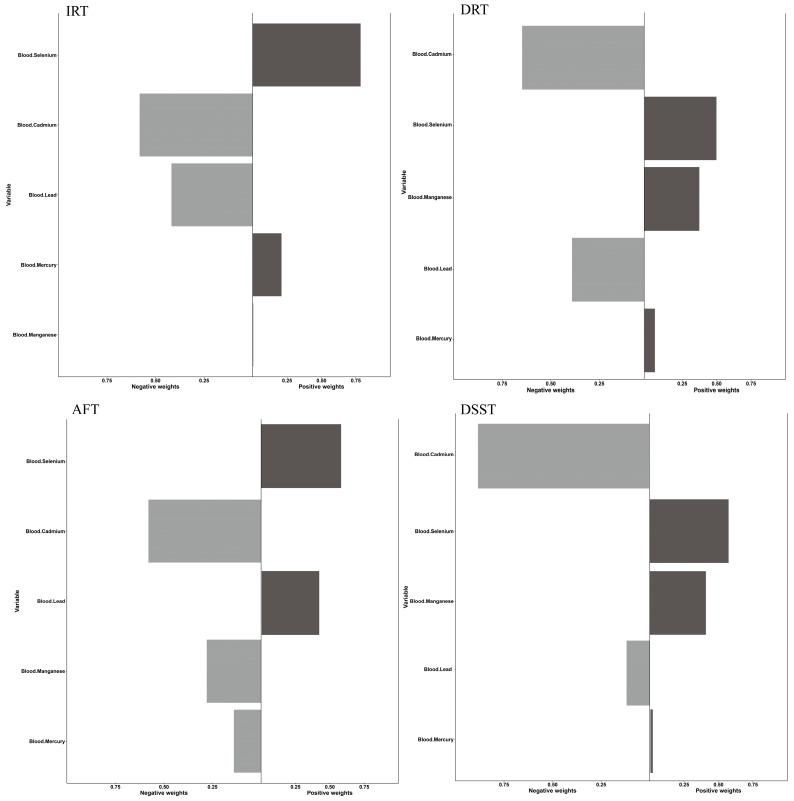
Qgcomp model regression index weights for blood heavy metals and four cognitive tests in older people. The Qgcomp models were adjusted by sex, age, race/ethnicity, education level, alcohol intake, and smoking status.

Compared to the WQS model, the Qgcomp model allows for direct evaluation of the effects of different input variables on the dependent variable as it refrains from making assertions about the combined impacts before testing. The findings of Figure S13 and Table S13 demonstrate that in the whole population, the cumulative effects of blood heavy metal levels exhibited a favorable tendency with DRT and DSST. In the male elderly population, a positive trend within blood heavy metal levels was connected with DRT and AFT; in the female population, a negative trend was associated with IRT and AFT; instead, a positive trend was associated with DSST. Blood cadmium had a significant negative impact on the DRT test findings (DRT: −0.56), but blood selenium had the most important positive effect in the IRT, AFT, and DSST tests in the male group (IRT: 0.95; AFT: 0.70; DSST: 0.83) (Figures S14–S17; Table S14). Results for the female group revealed that blood cadmium had the greatest negative effects in IRT, DRT, and DSST (IRT: −0.66; DRT: −0.78; DSST: 1.00), while a substantial positive impact of blood lead (AFT: 1.00) could be identified in the AFT test (Figure S17, Table S15). The combined impact of serum heavy metals in the evidence for the entire community was similar to the earlier findings (positive: blood selenium; negative: blood cadmium) (Figure 6, Table S15).

## 4. Discussion

Our study attempted to determine whether there is a relationship between cognitive function and blood heavy metals levels in older people and whether there are sex differences within this association. In this investigation, the GLM, BKMR, WQS, and Qgcomp models were utilized to evaluate the complex effects of five serum heavy metals on cognitive function. We identified a positive correlation between selenium levels in the blood and cognitive function in the elderly. Cadmium and lead were significantly and negatively associated with cognitive function. Men, on average, had weaker cognitive skills than women, according to studies that considered age into view. Moreover, both in the positive and negative related directions of the study, older male participants demonstrated a more marked reaction in cognitive capacity to blood heavy metals. Our main results imply that the negative correlation of other heavy metals with cognitive function may be reduced or even reversed by selenium. This study emphasizes the significance of determining the typical effects of metals in blood on cognitive function using various statistical techniques and contrasting the outcomes while considering the advantages and limitations of multiple methods.

Organs prone to metal ion enrichment and elevated metabolic activity include the nervous system in general and the brain in specific. Nerve deterioration and reactive stress may result from disturbances in metal balance [29]. This study discovered that in both monometallic and polymetallic models, selenium was favorably linked with brain performance. Due to its distinct neurophysiological characteristics and beneficial qualities, selenium is generally considered necessary [12,30]. The redox activity of metal elements and reactive stress constitute important biochemical signaling pathways for cognitive function [31]. Selenium, in the shape of selenoproteins, plays an assortment of roles in normal metabolic processes and metabolism. Glutathione peroxidase, a typical selenoprotein, has an antioxidant impact and may protect against cellular damage from reactive oxygen species [12]. Food sources are a substantial contribution to blood heavy metal levels [5], and dietary patterns have a considerable impact on serum elemental metal concentrations [28]. Through the correction of metabolic imbalances and the reduction of inflammation and oxidative stress, moderate selenium supplementation may enhance cognitive performance [32]. Notably, although the association of blood selenium on cognitive function was robust in this study, the strongest positive association was observed at moderate levels. Consideration should also be given to the health concerns associated with consuming too many foods high in selenium [33,34].

Concerns have been expressed with the increasing proportion of older individuals in the global population and their potential increased susceptibility to multiple metal exposures due to physiological factors [35]. According to this study, blood levels of lead and cadmium are negatively correlated with cognitive ability in the elderly. The same trends have been discovered in health investigations conducted in other areas [10,11]. People are continuously subjected to numerous elements at the exact moment rather than just one. Metals can have varying health impacts in situations of mixed exposure due to combined or adverse interactions [34,36]. Our core findings suggest that the protective effect of selenium might mitigate or even reverse the negative association between other heavy metals and cognitive function. All elements of the growth, operation, aging, and illness of the central nervous system depend on redox balance, and an imbalance causes neurodegeneration [31]. Redox homeostasis is readily impacted by abnormalities in metal homeostasis. Reduced selenium levels result in neural malfunction, which has been linked to gender in animal research [37]. Testes may make males more susceptible to the physiological effects of selenium antioxidants, offering neuroprotection [37]. According to several studies, oxidative stress caused by heavy metals like lead and cadmium causes a variety of physiological, biochemical, and behavioral dysfunctions in people [29,38,39]. This may be one of the possible mechanisms by which selenium counteracts the cognitive impairment associated with other heavy metals. We are, to our understanding, one of the few studies to have examined the impact of lead and cadmium, as well as selenium alone, on older people’s cognitive function. For the variables affecting this joint result to be confirmed, more in vivo research is required.

Subgroup studies offered distinctive perspectives on metal exposure in various groups. According to the results of the mixed exposure study, males and females may be affected differently by exposure to mixed metals; a more excellent relationship (positive or negative) between mixed metals and cognitive performance in males was also shown. The disparities in the bodily loads of metal buildup between men and women may explain these sex-specific correlations [40]. Selenium metabolism and expression of selenoproteins are sexually dimorphic [30]. The liver and kidney of male and female rats differ in producing the proteins selenoprotein GPx1, selenoprotein P, and iodothyronine deiodinase 1, with females producing more selenoprotein when given identical amounts of selenium [30]. Males have a more significant load of heavy metals and are more vulnerable to the deleterious impacts of heavy metals, including impaired cognitive development, focus impairments, and behavioral issues, according to epidemiological and laboratory research [40,41]. Neurotoxic effects that are particular to one sex may be caused by sex variations impacting these processes, such as differences in accumulation, antioxidant capacity, nutritional needs, sex hormones, and methylation/gene expression. There are several interrelated variables that impact metal-related neurotoxicity, ranging from molecular, hormonal, and epigenetic processes influencing gene regulation, expression, and function to sexually dimorphic changes in absorption and metabolism [14,17,40,42]. The relationship between mental and physical makeup may also partially contribute to sex variations in the cumulative impacts of heavy metals [15]. In addition, additional hypotheses, such as those involving endocrine, genetic, biochemical, structural, and environmental variables, may explain the sex-based unequal vulnerability [16,43].

Previous research on the effects of blood heavy metals on cognitive function has only used single pollutant models, which may result in biased impact evaluations due to the neglect of mixed effects [9,18]. Therefore, it is crucial to employ specialized techniques to investigate the mixed implications of multiple pollutants. Determining which statistical method is most appropriate for this research is challenging as there is no a priori information. To investigate the impact of serum heavy metals on cognitive function in the elderly, this study employed a variety of statistical models, including the Bayesian kernel machine regression (BKMR) model, the weighted quantile sum (WQS) regression model, and the Quantile g-computation (Qgcomp) regression model [34,44]. The findings will vary depending on particular approaches because these statistical techniques place varying emphasis on handling distinct statistical characteristics (high dimensionality, multicollinearity, interaction, and nonlinear effects). Combining different statistical approaches can help provide reasonably comprehensive information and avoids the one-sidedness of a single approach [45]. Nevertheless, additional study is required to address mixture and interaction effects using statistical methods and to clarify biological processes due to the intricacy of the real environmental effects of multiple metals.

The application of multiple statistical models in this study, which enabled us to thoroughly evaluate the relationship between metal mixtures and individual metals with cognitive abilities and ensure the validity of our results, is one of its major advantages. Additionally, we determined the weight between each metal pair metal mixture and cognitive ability, which was infrequently evaluated in earlier research. Moreover, NHANES is broadly representative, so our findings could be applied to various American groups. However, some limitations should be acknowledged. The data used in this research originates from a cross-sectional survey. There is no longitudinal follow-up of cognitive status nor of heavy metals concentrations, which means that a causal relationship cannot be envisaged, as there may be reverse causality. Additionally, a single measurement might not accurately reflect exposure over the day because each metal has a distinct distribution and half-life in the human body. The physiological operation of the brain depends on the homeostasis of metal ions [38]; through a variety of biochemical mechanisms, trace mineral components, including iron, copper, zinc, and manganese, may have an impact on brain health [43]. Base metals such as copper, zinc, and iron were not included in this study, which limits the usable scope of the findings. The chance of unmeasured factors distorting the outcomes cannot be ruled out, although important predictors were included in our model. The chance of unmeasured factors distorting the outcomes cannot be ruled out, such as covariates vascular risk factors, although important predictors were included in our model. Although we adjusted for potential confounders to the best of our ability, residual confounders are inevitable in the observation environment, including age, education, lifestyle habits, etc. BKMR, WQS, and Qgcomp do not currently have weight-based statistical analyses, which may impact the validity of the study results.

## 5. Conclusions

In conclusion, we discovered that blood selenium was positively correlated with cognitive performance in older people. In contrast, levels of cadmium and lead in the blood were negatively associated with cognitive ability. Selenium might partially mitigate or even reverse the negative correlation of heavy metal mixtures on cognitive function. In addition, a significant result of our research was that males performed cognitively, on average, worse than women. It appears that males are more susceptible to the impacts of exposure to mixed metals, either positively or unfavorably, and the relationship between mixed metals and cognitive function has been demonstrated to be stronger in men. These findings need to be confirmed by additional cohort studies, nevertheless, given the cross-sectional approach of our research.

## Figures and Tables

**Table 1:** Descriptive statistical results of serum heavy metal content in the population were included.

		Percentile						
	Mean	25th	50th	75th	Skew.2SE ^a^	Kurt.2SE ^b^	Normtest.W ^c^	Normtest.p
Blood lead	1.90	1.03	1.49	2.24	50.96	265.31	0.60	<0.001
Blood cadmium	0.55	0.27	0.40	0.65	23.98	43.02	0.72	<0.001
Blood mercury	1.87	0.56	1.04	2.11	42.98	176.52	0.57	<0.001
Blood selenium	195.25	177.90	193.50	208.30	52.00	364.67	0.72	<0.001
Blood manganese	9.48	7.04	8.79	11.18	37.83	177.17	0.75	<0.001

^a^ The skewness coefficient g1 (skewness), its significant criterium (skew. 2SE, that is, g1/2. SEg1; if skew. 2SE > 1, then skewness is significantly different than zero). ^b^ The kurtosis coefficient g2 (kurtosis), its significant criterium (kurt. 2SE, same remark than for skew.2SE). ^c^ The statistic of a Shapiro–Wilk test of normality (normtest.W) and its associated probability (normtest.p).

**Table 2:** Characteristics of the study population.

Characteristic	Overall, N = 1833 (100%) ^1^	Male, N = 883 (45%) ^1^	Female, N = 950 (55%) ^1^	*p*-Value ^2^
Age				0.031
60–69 years	921 (51%)	460 (55%)	461 (48%)	
70–79 years	516 (29%)	247 (28%)	269 (29%)	
80+ years	396 (20%)	176 (17%)	220 (23%)	
Race/ethnicity				0.200
Non-Hispanic White	862 (80%)	389 (80%)	473 (81%)	
Non-Hispanic Black	436 (7.8%)	220 (7.2%)	216 (8.4%)	
Other Hispanic	203 (3.9%)	100 (4.0%)	103 (3.9%)	
Other/Multi-Racial	166 (3.5%)	82 (3.4%)	84 (3.5%)	
Mexican American	138 (2.9%)	74 (3.2%)	64 (2.6%)	
Other Race, Including Multi-Racial	28 (1.7%)	18 (2.2%)	10 (0.6%)	
Education				<0.001
Less Than 9th Grade	219 (6.0%)	124 (7.0%)	95 (5.1%)	
9–11th Grade	246 (9.4%)	116 (9.4%)	130 (9.3%)	
High School Grad/GED	421 (22%)	188 (18%)	233 (25%)	
Some College or AA degree	514 (31%)	222 (27%)	292 (34%)	
College Graduate or above	433 (32%)	233 (39%)	200 (27%)	
Smoking status				<0.001
Never smoker	894 (48%)	297 (34%)	597 (60%)	
Current smoker	234 (11%)	147 (13%)	87 (8.3%)	
Former smoker	705 (41%)	439 (53%)	266 (31%)	
Alcohol intake				<0.001
1–5 drinks/month	864 (44%)	497 (49%)	367 (40%)	
5–10 drinks/month	89 (6.5%)	51 (6.9%)	38 (6.1%)	
10+ drinks/month	307 (23%)	198 (31%)	109 (16%)	
Non-drinker	573 (27%)	137 (13%)	436 (38%)	
IRT	20.0 (17.0, 23.0)	19.0 (16.0, 22.0)	21.0 (17.0, 23.0)	<0.001
DRT	6.00 (5.00, 8.00)	6.00 (4.00, 7.00)	7.00 (5.00, 8.00)	<0.001
AFT	18.0 (14.0, 22.0)	19.0 (14.0, 22.0)	18.0 (14.0, 22.0)	0.100
DSST	54 (42, 65)	50 (40, 62)	56 (43, 67)	<0.001

Notes: ^1^ n (unweighted) (weighted%); Median (IQR). ^2^ chi-squared tests with Rao and Scott’s second-order correction; Wilcoxon rank-sum test for complex survey samples.

**Table 3:** Summary results from BKMR and Qgcomp analysis in the whole population.

Variable	BKMR PIP				Qgcomp			
	IRT	DRT	AFT	DSST	IRT	DRT	AFT	DSST
Blood Lead	0.15	0.29	0.26	0.49	−0.42	−0.37	0.42	−0.12
Blood Cadmium	0.36	0.37	0.33	0.83	−0.58	−0.63	−0.58	−0.88
Blood Mercury	0.05	0.17	0.06	0.21	0.21	0.08	−0.14	0.02
Blood Selenium	0.99	0.94	0.47	1.00	0.78	0.52	0.58	0.57
Blood Manganese	0.21	0.75	0.25	0.93	0.054	0.40	−0.28	0.41

Models adjusted for gender, age, race/ethnicity, education level, alcohol intake, and smoking status.

**Table 4:** Summary results from WQS analysis in the whole population.

Variable	IRT Positive	IRT Negative	DRT Positive	DRT Negative	AFT Positive	AFT Negative	DSST Positive	DSST Negative
Blood Selenium	0.94	0.00	0.68	0.02	0.80	0.00	0.82	0.00
Blood Manganese	0.01	0.34	0.25	0.02	0.04	0.17	0.14	0.01
Blood Mercury	0.04	0.02	0.04	0.07	0.08	0.04	0.03	0.07
Blood Lead	0.00	0.38	0.01	0.41	0.06	0.12	0.00	0.40
Blood Cadmium	0.00	0.25	0.02	0.48	0.02	0.66	0.00	0.52

Models adjusted for gender, age, race/ethnicity, education level, alcohol intake, and smoking status.

## Data Availability

The data that support the findings of this study are openly available in NHANES at https://wwwn.cdc.gov/Nchs/Nhvanes/ (accessed on 1 February 2023). Further inquiries can be directed to the corresponding author.

## Supplementary Materials

The following supporting information can be downloaded at https://www.mdpi.com/article/10.3390/nu15132874/s1. Table S1. Associations of single blood heavy metals and cognitive function on elderly. Table S2. Associations of single blood heavy metals and cognitive function on male elderly. Table S3. Associations of single blood heavy metals and cognitive function on female elderly. Table S4. Associations of single blood heavy metals (quartile conversion) and cognitive function on elderly. Table S5. Associations of single blood heavy metals (quartile conversion) and cognitive function on male elderly. Table S6. Associations of single blood heavy metals (quartile conversion) and cognitive function on female elderly. Table S7. Summary results from BKMR analysis in the whole population. Table S8. Summary results from BKMR analysis in males. Table S9. Summary results from BKMR analysis in females. Table S10. Summary results from WQS analysis in the whole population. Table S11. Summary results from WQS analysis in males. Table S12. Summary results from WQS analysis in females. Table S13. Summary results from Qgcomp analysis in the whole population. Table S14. Summary results from Qgcomp analysis in males. Table S15. Summary results from Qgcomp analysis in females. Figure S1. Spearman correlation plot of concentrations of individual metals in older people. Figure S2. Spearman correlation plot of concentrations of individual metals in males. Figure S3. Spearman correlation plot of concentrations of individual metals in females. Figure S4. Combined effects of the metal as a mixture on cognitive function in males. Figure S5. Combined effects of the metal as a mixture on cognitive function in females. Figure S6. Univariate exposure–response functions and 95% confidence interval for each heavy metal with the other metals fixed at the median in males. Models adjusted for age, race/ethnicity, education level, alcohol intake, and smoking status. Figure S7. Univariate exposure–response functions and 95% confidence interval for each heavy metal with the other metals fixed at the median in females. Models adjusted for age, race/ethnicity, education level, alcohol intake, and smoking status. Figure S8. Estimated effects (95% CIs) of single aldehydes on cognitive function of elderly people when the levels of other aldehydes were fixed at 25th, 50th, and 75th. Models adjusted for sex, age, race/ethnicity, education level, alcohol intake, and smoking status. CI, confidence interval. Figure S9. Estimated effects (95% CIs) of single aldehyde on cognitive function of males when the levels of other aldehydes were fixed at 25th, 50th, and 75th. Models adjusted for age, race/ethnicity, education level, alcohol intake, and smoking status. CI, confidence interval. Figure S10. Estimated effects (95% CIs) of single aldehydes on cognitive function of females when the levels of other aldehydes were fixed at 25th, 50th, and 75th. Models adjusted for age, race/ethnicity, education level, alcohol intake, and smoking status. CI, confidence interval. Figure S11. WQS model regression index weights for a total of four cognitive tests in males. The WQS models were adjusted by age, race/ethnicity, education level, alcohol intake, and smoking status. Figure S12. WQS model regression index weights for a total of four cognitive tests in females. The WQS models were adjusted by age, race/ethnicity, education level, alcohol intake, and smoking status. Figure S13. Qgcomp model regression joint effect (95% CI) for blood heavy metals and four cognitive tests in older people. The Qgcomp models were adjusted by sex, age, race/ethnicity, education level, alcohol intake, and smoking status. Figure S14. Qgcomp model regression joint effect (95% CI) for blood heavy metals and four cognitive tests in males. The Qgcomp models were adjusted by age, race/ethnicity, education level, alcohol intake, and smoking status. Figure S15. Qgcomp model regression joint effect (95% CI) for blood heavy metals and four cognitive tests in females. The Qgcomp models were adjusted by age, race/ethnicity, education level, alcohol intake, and smoking status. Figure S16. Qgcomp model regression index weights for blood heavy metals and four cognitive tests in males. The Qgcomp models were adjusted by age, race/ethnicity, education level, alcohol intake, and smoking status. Figure S17. Qgcomp model regression index weights for blood heavy metals and four cognitive tests in females. The Qgcomp models were adjusted by age, race/ethnicity, education level, alcohol intake, and smoking status.
