# Supplementary material for: Sex Specificity in the Mixed Effects of Blood Heavy Metals and Cognitive Function on Elderly: Evidence from NHANES
Source: Nutrients. 2023 Jun 25;15(13):2874. doi: 10.3390/nu15132874 (PMC10343838; doi:10.3390/nu15132874)

Sex Specificity in the mixed effects of blood heavy metals and cognitive function on elderly: Evidence from NHANES

Supplementary Materials

CONTENTS

Table S1. Associations of single blood heavy metals and cognitive function on elderly. 1
Table S2. Associations of single blood heavy metals and cognitive function on male elderly. 1
Table S3. Associations of single blood heavy metals and cognitive function on female elderly. 2
Table S4. Associations of single blood heavy metals (quartile conversion) and cognitive function on elderly. 2
Table S5. Associations of single blood heavy metals (quartile conversion) and cognitive function on male elderly. 4
Table S6. Associations of single blood heavy metals (quartile conversion) and cognitive function on female elderly. 5
Table S7. Summary results from BKMR analysis in the whole population. 7
Table S8. Summary results from BKMR analysis in males. 7
Table S9. Summary results from BKMR analysis in females. 8
Table S10. Summary results from WQS analysis in the whole population. 8
Table S11. Summary results from WQS analysis in males. 8
Table S12. Summary results from WQS analysis in females. 9
Table S13. Summary results from Qgcomp analysis in the whole population. 9
Table S14. Summary results from Qgcomp analysis in males. 9
Table S15. Summary results from Qgcomp analysis in females. 10
Figure S1. Spearman correlation plot of concentrations of individual metals in older people. 11

Figure S2. Spearman correlation plot of concentrations of individual metals in males. 12
Figure S3. Spearman correlation plot of concentrations of individual metals in females. 13
Figure S4. Combined effects of the metal as a mixture on cognitive function in males. 14
Figure S5. Combined effects of the metal as a mixture on cognitive function in females. 15
Figure S6. Univariate exposure-response functions and 95% confidence interval for each heavy metal with the other metals fixed at the median in males. Models adjusted for age, race/ethnicity, education level, alcohol intake, smoking status. 16
Figure S7. Univariate exposure-response functions and 95% confidence interval for each heavy metal with the other metals fixed at the median in females. Models adjusted for, age, race/ethnicity, education level, alcohol intake, smoking status. 17
Figure S8. Estimated effects (95% CIs) of single aldehyde on cognitive function of elderly people when the levels of other aldehydes were fixing at 25th, 50th, and 75th. Models adjusted for sex, age, race/ethnicity, education level, alcohol intake, smoking status. CI, confidence interval. 18
Figure S9. Estimated effects (95% CIs) of single aldehyde on cognitive function of males when the levels of other aldehydes were fixing at 25th, 50th, and 75th. Models adjusted for age, race/ethnicity, education level, alcohol intake, smoking status. CI, confidence interval. 19
Figure S10. Estimated effects (95% CIs) of single aldehyde on cognitive function of females when the levels of other aldehydes were fixing at 25th, 50th, and 75th. Models adjusted for age, race/ethnicity, education level, alcohol intake, smoking status. CI, confidence interval. 20
Figure S11. WQS model regression index weights for total four cognitive tests in males. The WQS models were adjusted by age, race/ethnicity, education level, alcohol intake, smoking status. 21
Figure S12. WQS model regression index weights for total four cognitive tests in females. The WQS models were adjusted by age, race/ethnicity, education level, alcohol intake, smoking status. 22
Figure S13. Qgcomp model regression joint effect (95% CI) for blood heavy metals and four cognitive tests in older people. The Qgcomp models were adjusted by sex, age, race/ethnicity, education level, alcohol intake, smoking status. 23
Figure S14. Qgcomp model regression joint effect (95% CI) for blood heavy metals and four cognitive tests in males. The Qgcomp models were adjusted by, age, race/ethnicity, education level, alcohol intake, smoking status. 24
Figure S15. Qgcomp model regression joint effect (95% CI) for blood heavy metals and four cognitive tests in females. The Qgcomp models were adjusted by, age, race/ethnicity, education level, alcohol intake, smoking status. 25

Figure S16. Qgcomp model regression index weights for blood heavy metals and four cognitive tests in males. The Qgcomp models were adjusted by age, race/ethnicity, education level, alcohol intake, smoking status. 26

Figure S17. Qgcomp model regression index weights for blood heavy metals and four cognitive tests in females. The Qgcomp models were adjusted by age, race/ethnicity, education level, alcohol intake, smoking status. 27

Table S1. Associations of single blood heavy metals and cognitive function on elderly.

	IRT			DRT			AFT			DSST		
Characteristic	Beta	95% CI1	*p*-value	Beta	95% CI1	*p*-value	Beta	95% CI1	*p*-value	Beta	95% CI1	p-value
Blood lead	-0.05	-0.21, 0.10	0.4	-0.02	-0.11, 0.07	0.6	-0.09	-0.21, 0.04	0.11	-0.52	-0.93, -0.11	**0.005**
Blood cadmium	-0.73	-1.5, 0.03	**0.033**	-0.30	-0.70, 0.11	0.10	-0.73	-1.8, 0.35	0.13	-3.0	-5.1, -0.99	**<0.001**
Blood mercury	0.03	-0.10, 0.15	0.6	-0.01	-0.06, 0.04	0.7	0.07	-0.11, 0.24	0.4	0.13	-0.23, 0.49	0.4
Blood selenium	0.01	0.00, 0.01	**<0.001**	0.00	0.00, 0.01	0.4	0.00	0.00, 0.01	0.3	0.01	-0.01, 0.04	0.3
Blood manganese	-0.04	-0.10, 0.02	0.14	0.00	-0.03, 0.03	>0.9	-0.05	-0.11, <0.001	**0.041**	0.00	-0.33, 0.32	>0.9

[1]CI = Confidence Interval
Models adjusted for sex, age, race/ethnicity, education level, alcohol intake, smoking status.

Table S2. Associations of single blood heavy metals and cognitive function on male elderly.

	**IRT**			**DRT**			**AFT**			**DSST**		
**Characteristic**	**Beta**	**95% CI[1]**	***p*-value**	**Beta**	**95% CI[1]**	***p*-value**	**Beta**	**95% CI[1]**	***p*-value**	**Beta**	**95% CI[1]**	***p*-value**
Blood lead	-0.02	-0.16, 0.12	0.7	-0.01	-0.11, 0.09	0.8	-0.07	-0.23, 0.08	0.3	-0.51	-0.80, -0.22	**<0.001**
Blood cadmium	-0.39	-1.5, 0.78	0.5	-0.17	-0.86, 0.51	0.6	-0.49	-2.1, 1.1	0.5	-2.0	-5.0, 1.0	0.14
Blood mercury	0.00	-0.18, 0.17	>0.9	0.00	-0.07, 0.06	0.9	0.16	-0.09, 0.40	0.2	0.06	-0.32, 0.43	0.7
Blood selenium	0.01	0.00, 0.02	**0.009**	0.00	-0.01, 0.01	>0.9	0.01	0.00, 0.02	0.10	0.01	-0.04, 0.05	0.7
Blood manganese	-0.04	-0.14, 0.05	0.3	0.00	-0.06, 0.05	0.8	-0.08	-0.18, 0.01	0.059	-0.37	-0.80, 0.05	0.052

[1]CI = Confidence Interval
Models adjusted for age, race/ethnicity, education level, alcohol intake, smoking status.

Table S3. Associations of single blood heavy metals and cognitive function on female elderly.

	IRT			DRT			AFT			DSST		
**Characteristic**	**Beta**	**95% CI**[1]	***p*-value**	**Beta**	**95% CI**[1]	***p*-value**	**Beta**	**95% CI**[1]	***p*-value**	**Beta**	**95% CI**[1]	***p*-value**
Blood lead	-0.20	-0.60, 0.20	0.3	-0.08	-0.32, 0.16	0.5	0.01	-0.44, 0.46	>0.9	-0.24	-1.7, 1.2	0.7
Blood cadmium	-1.1	-2.0, -0.10	**0.015**	-0.35	-0.82, 0.12	0.10	-0.92	-2.3, 0.52	0.2	-4.0	-7.2, -0.92	**0.004**
Blood mercury	0.06	-0.06, 0.18	0.3	-0.01	-0.08, 0.06	0.7	-0.03	-0.25, 0.19	0.8	0.20	-0.26, 0.66	0.3
Blood selenium	0.00	0.00, 0.01	0.12	0.00	0.00, 0.01	0.2	0.00	-0.01, 0.01	0.8	0.01	-0.01, 0.04	0.2
Blood manganese	-0.04	-0.12, 0.04	0.3	0.00	-0.05, 0.04	>0.9	-0.03	-0.11, 0.05	0.5	0.25	-0.14, 0.64	0.2

[1]CI = Confidence Interval

Models adjusted for sex, age, race/ethnicity, education level, alcohol intake, smoking status.

Table S4. Associations of single blood heavy metals (quartile conversion) and cognitive function on elderly.

	IRT			DRT			AFT			DSST		
**Characteristic**	**Beta**	**95% CI**[1]	***p*-value**	**Beta**	**95% CI**[1]	***p*-value**	**Beta**	**95% CI**[1]	***p*-value**	**Beta**	**95% CI**[1]	***p*-value**
Blood lead			0.3			0.6			0.2			0.4
Q1	—	—		—	—		—	—		—	—	
Q2	-0.09	-0.79, 0.60		-0.15	-0.57, 0.27		-0.30	-1.5, 0.90		0.74	-1.8, 3.3	
Q3	-0.57	-1.3, 0.18		-0.21	-0.61, 0.20		0.32	-0.43, 1.1		0.93	-2.1, 4.0	
Q4	-0.32	-1.3, 0.66		-0.12	-0.59, 0.35		-0.05	-1.0, 0.90		-1.2	-5.3, 2.9	
Blood cadmium			**0.028**			**<0.001**			0.2			0.086
Q1	—	—		—	—		—	—		—	—	

	IRT			DRT			AFT			DSST		
**Characteristic**	**Beta**	**95% CI[1]**	***p*-value**	**Beta**	**95% CI[1]**	***p*-value**	**Beta**	**95% CI[1]**	***p*-value**	**Beta**	**95% CI[1]**	***p*-value**
Q2	-0.21	-1.0, 0.59		0.06	-0.47, 0.60		0.29	-0.71, 1.3		-1.1	-4.4, 2.2	
Q3	-0.50	-1.3, 0.27		-0.60	-1.0, -0.17		-0.11	-1.0, 0.79		-1.8	-4.2, 0.67	
Q4	-0.97	-1.8, -0.17		-0.46	-0.92, 0.00		-0.67	-1.7, 0.37		-2.9	-5.9, 0.04	
Blood mercury			0.7			0.6			0.6			0.040
Q1	—	—		—	—		—	—		—	—	
Q2	0.32	-0.61, 1.3		0.04	-0.37, 0.44		0.33	-0.77, 1.4		0.88	-2.0, 3.8	
Q3	0.54	-0.49, 1.6		0.08	-0.41, 0.56		0.18	-0.54, 0.91		1.1	-1.1, 3.3	
Q4	0.44	-0.59, 1.5		0.23	-0.21, 0.66		0.64	-0.46, 1.7		2.5	0.39, 4.6	
Blood selenium			**0.009**			**0.079**			**0.013**			**<0.001**
Q1	—	—		—	—		—	—		—	—	
Q2	1.4	0.41, 2.3		0.47	0.02, 0.92		1.0	0.20, 1.8		4.1	1.5, 6.8	
Q3	0.63	-0.42, 1.7		0.28	-0.22, 0.77		0.59	-0.28, 1.5		3.1	1.1, 5.2	
Q4	0.81	-0.04, 1.7		0.30	-0.07, 0.68		0.83	-0.14, 1.8		3.2	0.54, 5.9	
Blood manganese			0.9			>0.9			0.6			0.8
Q1	—	—		—	—		—	—		—	—	
Q2	0.07	-0.59, 0.74		0.06	-0.25, 0.37		-0.22	-1.5, 1.1		0.72	-1.8, 3.3	
Q3	0.00	-0.75, 0.75		0.02	-0.32, 0.36		-0.38	-1.4, 0.65		0.53	-2.1, 3.2	
Q4	-0.21	-1.0, 0.62		0.02	-0.36, 0.40		-0.54	-1.6, 0.47		1.1	-1.5, 3.8	

[1]CI = Confidence Interval

Characteristic	IRT Beta	IRT 95% CI[1]	IRT *p*-value	DRT Beta	DRT 95% CI[1]	DRT *p*-value	AFT Beta	AFT 95% CI[1]	AFT *p*-value	DSST Beta	DSST 95% CI[1]	DSST *p*-value

Models adjusted for sex, age, race/ethnicity, education level, alcohol intake, smoking status.

Table S5. Associations of single blood heavy metals (quartile conversion) and cognitive function on male elderly.

	IRT			DRT			AFT			DSST		
**Characteristic**	**Beta**	**95% CI[1]**	***p*-value**	**Beta**	**95% CI[1]**	***p*-value**	**Beta**	**95% CI[1]**	***p*-value**	**Beta**	**95% CI[1]**	***p*-value**
Blood lead			0.3			0.9			0.9			0.3
Q1	—	—		—	—		—	—		—	—	
Q2	-0.20	-1.4, 0.96		-0.19	-1.1, 0.67		-0.19	-1.1, 0.67		-0.81	-4.1, 2.5	
Q3	-1.0	-2.4, 0.33		-0.19	-0.99, 0.60		-0.19	-0.99, 0.60		-1.7	-5.8, 2.4	
Q4	-0.57	-2.1, 0.94		-0.07	-0.83, 0.70		-0.07	-0.83, 0.70		-3.4	-7.8, 0.91	
Blood cadmium			0.4			**0.021**			0.3			0.2
Q1	—	—		—	—		—	—		—	—	
Q2	-0.16	-1.3, 1.0		0.31	-0.46, 1.1		0.90	-0.64, 2.4		-1.4	-4.8, 2.1	
Q3	-0.43	-1.7, 0.79		-0.58	-1.3, 0.19		0.03	-1.2, 1.3		-3.0	-6.6, 0.57	
Q4	-0.73	-1.8, 0.34		-0.10	-0.67, 0.48		-0.76	-2.3, 0.82		-2.3	-6.3, 1.6	
Blood mercury			0.11			>0.9			0.5			0.5
Q1	—	—		—	—		—	—		—	—	
Q2	1.3	0.05, 2.5		0.08	-0.41, 0.57		0.55	-0.90, 2.0		-0.45	-2.9, 2.0	
Q3	0.87	-0.37, 2.1		0.14	-0.45, 0.74		0.01	-1.7, 1.7		0.03	-4.0, 4.1	
Q4	0.41	-1.1, 1.9		0.09	-0.54, 0.72		0.95	-1.1, 3.0		1.4	-1.7, 4.5	

Characteristic	IRT			DRT			AFT			DSST		
	Beta	95% CI[1]	*p*-value	Beta	95% CI[1]	*p*-value	Beta	95% CI[1]	*p*-value	Beta	95% CI[1]	*p*-value
Blood selenium			**0.022**			0.2			**0.047**			**0.013**
Q1	—	—		—	—		—	—		—	—	
Q2	1.6	0.34, 2.8		0.55	-0.04, 1.1		1.1	-0.50, 2.8		5.6	1.4, 9.7	
Q3	0.69	-0.69, 2.1		0.37	-0.39, 1.1		1.1	-0.33, 2.6		3.4	-0.39, 7.2	
Q4	0.89	-0.35, 2.1		0.27	-0.25, 0.79		1.8	0.26, 3.3		4.1	0.79, 7.4	
Blood manganese			0.8			>0.9			0.2			0.4
Q1	—	—		—	—		—	—		—	—	
Q2	-0.38	-1.4, 0.68		0.08	-0.41, 0.57		-0.86	-2.6, 0.88		-1.0	-4.8, 2.7	
Q3	-0.27	-1.5, 0.94		0.14	-0.45, 0.74		-0.55	-2.2, 1.1		0.00	-3.7, 3.7	
Q4	-0.46	-1.7, 0.72		0.09	-0.54, 0.72		-1.2	-2.5, 0.14		-2.3	-5.6, 0.94	

[1]CI = Confidence Interval

Models adjusted for age, race/ethnicity, education level, alcohol intake, smoking status.

Table S6. Associations of single blood heavy metals (quartile conversion) and cognitive function on female elderly.

Characteristic	IRT			DRT			AFT			DSST		
	Beta	95% CI[1]	*p*-value	Beta	95% CI[1]	*p*-value	Beta	95% CI[1]	*p*-value	Beta	95% CI[1]	*p*-value
Blood lead			>0.9			>0.9			0.12			0.6
Q1	—	—		—	—		—	—		—	—	
Q2	-0.08	-0.99, 0.83		-0.08	-0.99, 0.83		-0.57	-2.0, 0.83		1.3	-2.1, 4.7	
Q3	-0.32	-1.3, 0.66		-0.32	-1.3, 0.66		0.71	-0.44, 1.9		2.2	-1.9, 6.3	

	IRT			DRT			AFT			DSST		
Characteristic	Beta	95% CI[1]	*p*-value	Beta	95% CI[1]	*p*-value	Beta	95% CI[1]	*p*-value	Beta	95% CI[1]	*p*-value
Q4	-0.22	-1.4, 0.99		-0.22	-1.4, 0.99		-0.20	-1.5, 1.1		-0.09	-5.6, 5.4	
Blood cadmium			0.2			0.2			0.8			0.2
Q1	—	—		—	—		—	—		—	—	
Q2	-0.16	-1.4, 1.0		-0.16	-1.4, 1.0		-0.15	-1.4, 1.1		-0.89	-5.7, 3.9	
Q3	-0.36	-1.4, 0.64		-0.36	-1.4, 0.64		-0.22	-1.4, 0.93		-0.59	-3.5, 2.4	
Q4	-1.1	-2.5, 0.26		-1.1	-2.5, 0.26		-0.59	-2.1, 0.90		-3.3	-7.2, 0.69	
Blood mercury			0.14			0.6			>0.9			0.079
Q1	—	—		—	—		—	—		—	—	
Q2	-0.50	-1.6, 0.61		-0.19	-0.69, 0.31		-0.03	-1.3, 1.2		1.6	-2.5, 5.7	
Q3	0.26	-0.92, 1.4		-0.02	-0.50, 0.46		0.29	-1.1, 1.7		1.5	-1.2, 4.3	
Q4	0.53	-0.45, 1.5		0.10	-0.34, 0.53		0.32	-1.2, 1.8		3.3	0.25, 6.4	
Blood selenium			**0.037**			0.3			0.3			**0.022**
Q1	—	—		—	—		—	—		—	—	
Q2	1.2	0.24, 2.2		0.47	-0.07, 1.0		0.87	-0.55, 2.3		3.6	0.83, 6.4	
Q3	0.57	-0.66, 1.8		0.20	-0.39, 0.80		0.11	-1.2, 1.4		2.9	0.03, 5.7	
Q4	0.64	-0.52, 1.8		0.33	-0.23, 0.90		-0.09	-1.5, 1.3		2.3	-1.2, 5.9	
Blood manganese			0.9			>0.9			0.9			0.13
Q1	—	—		—	—		—	—		—	—	
Q2	0.34	-0.79, 1.5		0.06	-0.44, 0.55		0.44	-0.94, 1.8		2.5	-1.3, 6.4	

Characteristic	IRT			DRT			AFT			DSST		
	Beta	95% CI[1]	*p*-value	Beta	95% CI[1]	*p*-value	Beta	95% CI[1]	*p*-value	Beta	95% CI[1]	*p*-value
Q3	0.18	-0.77, 1.1		-0.05	-0.43, 0.34		0.09	-0.91, 1.1		1.4	-2.0, 4.9	
Q4	-0.04	-1.1, 1.0		-0.05	-0.69, 0.60		0.04	-1.4, 1.5		3.9	0.11, 7.6	

[1]CI = Confidence Interval

Models adjusted for age, race/ethnicity, education level, alcohol intake, smoking status.

Table S7. Summary results from BKMR analysis in the whole population.

Variable	BKMR PIP			
	IRT	DRT	AFT	DSST
Blood Lead	0.15	0.29	0.26	0.49
Blood Cadmium	0.36	0.37	0.33	0.83
Blood Mercury	0.05	0.17	0.06	0.21
Blood Selenium	0.99	0.94	0.47	1.00
Blood Manganese	0.21	0.75	0.25	0.93

Table S8. Summary results from BKMR analysis in males.

Variable	IRT	DRT	AFT	DSST
Blood Lead	0.075	0.38	0.176	0.47
Blood Cadmium	0.158	0.33	0.192	0.56
Blood Mercury	0.029	0.15	0.046	0.14
Blood Selenium	0.987	0.71	0.814	0.99
Blood Manganese	0.115	0.86	0.087	0.43

Table S9. Summary results from BKMR analysis in females.

Variable	IRT	DRT	AFT	DSST
Blood Lead	0.16	0.14	0.19	0.53
Blood Cadmium	0.44	0.24	0.30	0.57
Blood Mercury	0.19	0.15	0.11	0.24
Blood Selenium	0.56	0.63	0.25	0.71
Blood Manganese	0.24	0.21	0.34	0.90

Table S10. Summary results from WQS analysis in the whole population.

Variable	IRT Positive	IRT Negative	DRT Positive	DRT Negative	AFT Positive	AFT Negative	DSST Positive	DSST Negative
Blood Selenium	0.94	0.00	0.68	0.02	0.80	0.00	0.82	0.00
Blood Manganese	0.01	0.34	0.25	0.02	0.04	0.17	0.14	0.01
Blood Mercury	0.04	0.02	0.04	0.07	0.08	0.04	0.03	0.07
Blood Lead	0.00	0.38	0.01	0.41	0.06	0.12	0.00	0.40
Blood Cadmium	0.00	0.25	0.02	0.48	0.02	0.66	0.00	0.52

Models adjusted for sex, age, race/ethnicity, education level, alcohol intake, smoking status.

Table S11. Summary results from WQS analysis in males.

Mix_name	IRT (+)	IRT (-)	DRT (+)	DRT (-)	AFT (+)	AFT (-)	DSST (+)	DSST (-)
Blood Selenium	0.00	0.00	0.60	0.02	0.70	0.01	0.82	0.01
Blood Manganese	0.08	0.34	0.38	0.00	007	0.15	0.11	0.10
Blood Mercury	0.32	0.02	0.00	0.14	0.11	0.03	0.02	0.10

Blood Lead	0.23	0.38	0.00	0.37	0.10	0.08	0.04	0.11
Blood Cadmium	0.03	0.36	0.02	0.46	0.01	0.72	0.01	0.69

Table S12. Summary results from WQS analysis in females.

Mix_name	IRT (+)	IRT (-)	DRT (+)	DRT (-)	AFT (+)	AFT (-)	DSST (+)	DSST (-)
Blood Selenium	0.50	0.06	0.70	0.01	0	0.08	0.68	0.00
Blood Manganese	0.34	0.04	0.18	0.04	0	0.05	0.23	0.00
Blood Mercury	0.02	0.18	0.05	0.07	0	0.29	0.01	0.20
Blood Lead	0.12	0.21	0.06	0.23	1	0.05	0.04	0.29
Blood Cadmium	0.02	0.51	0.00	0.64	0	0.54	0.04	0.51

Table S13. Summary results from Qgcomp analysis in the whole population.

Variable	IRT	IRT	DRT	AFT	DSST
Blood Lead	0.15	-0.42	-0.37	0.42	-0.12
Blood Cadmium	0.36	-0.58	-0.63	-0.58	-0.88
Blood Mercury	0.05	0.21	0.08	-0.14	0.02
Blood Selenium	0.99	0.78	0.52	0.58	0.57
Blood Manganese	0.21	0.054	0.40	-0.28	0.41

Table S14. Summary results from Qgcomp analysis in males.

Mix_name	IRT	DRT	AFT	DSST
Blood Selenium	0.95	0.52	0.70	0.83

Blood Manganese	0.051	0.48	-0.30	0.17
Blood Mercury	-0.15	-0.08	-0.28	-0.052
Blood Lead	-0.31	-0.36	0.30	-0.44
Blood Cadmium	-0.55	-0.56	-0.42	-0.51

Table S15. Summary results from Qgcomp analysis in females.

Mix_name	IRT	DRT	AFT	DSST
Blood Selenium	0.41	0.52	-0.30	0.29
Blood Manganese	-0.16	0.23	-0.023	0.43
Blood Mercury	0.59	0.26	-0.022	0.045
Blood Lead	-0.18	-0.22	1	0.24
Blood Cadmium	-0.66	-0.78	-0.66	1

Figure S1. Spearman correlation plot of concentrations of individual metals in older people.

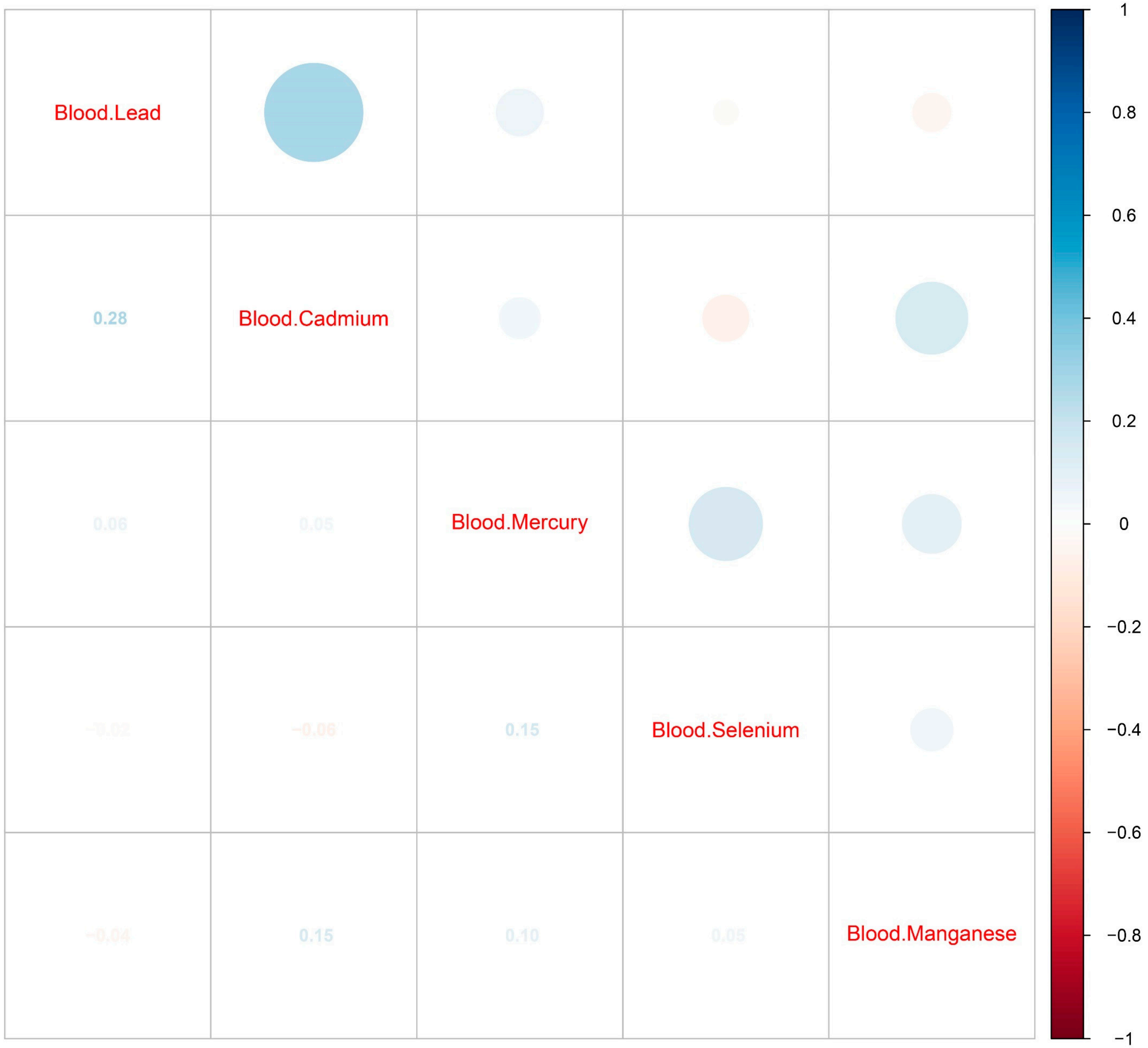

Figure S2. Spearman correlation plot of concentrations of individual metals in males.

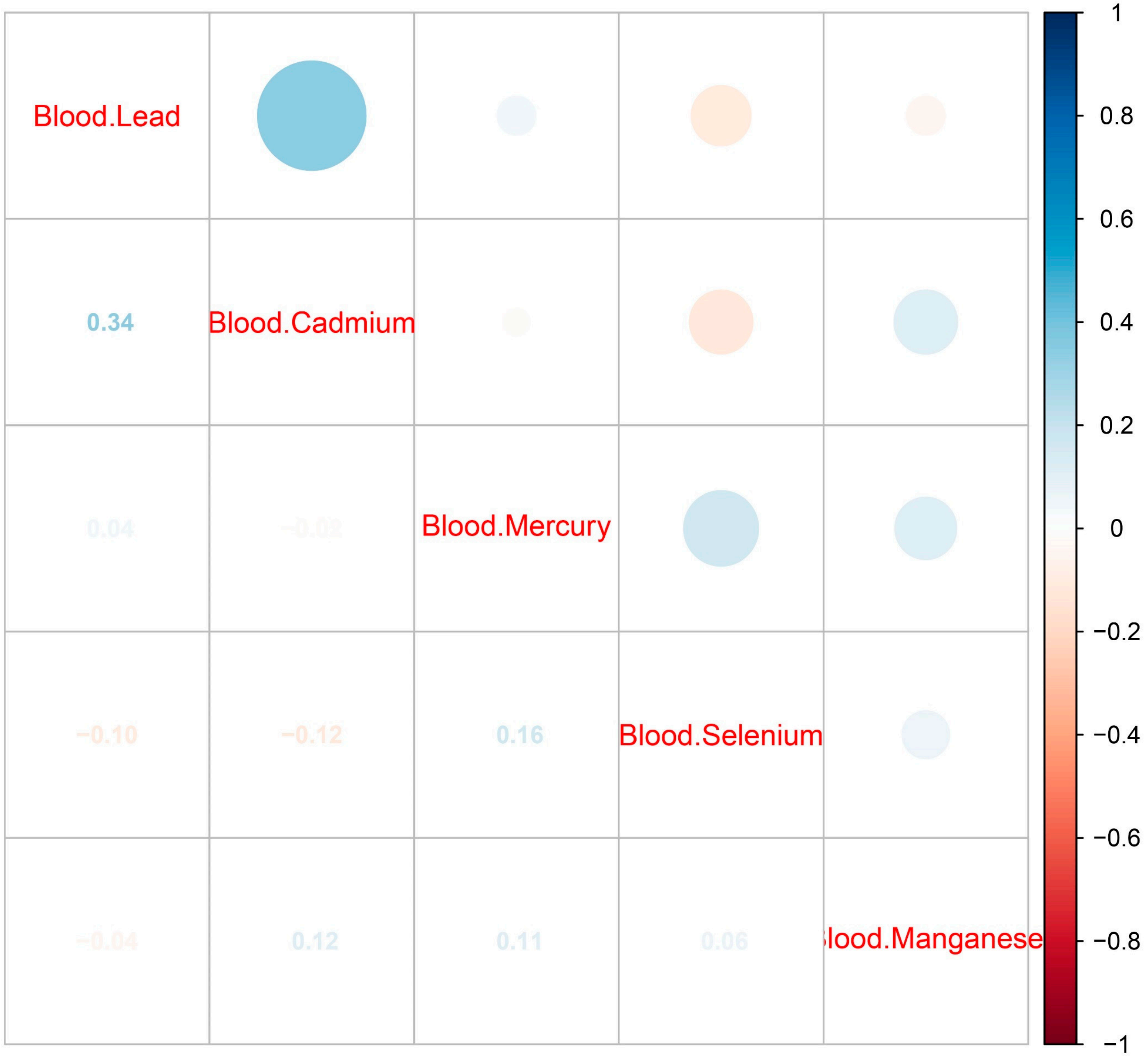

Figure S3. Spearman correlation plot of concentrations of individual metals in females.

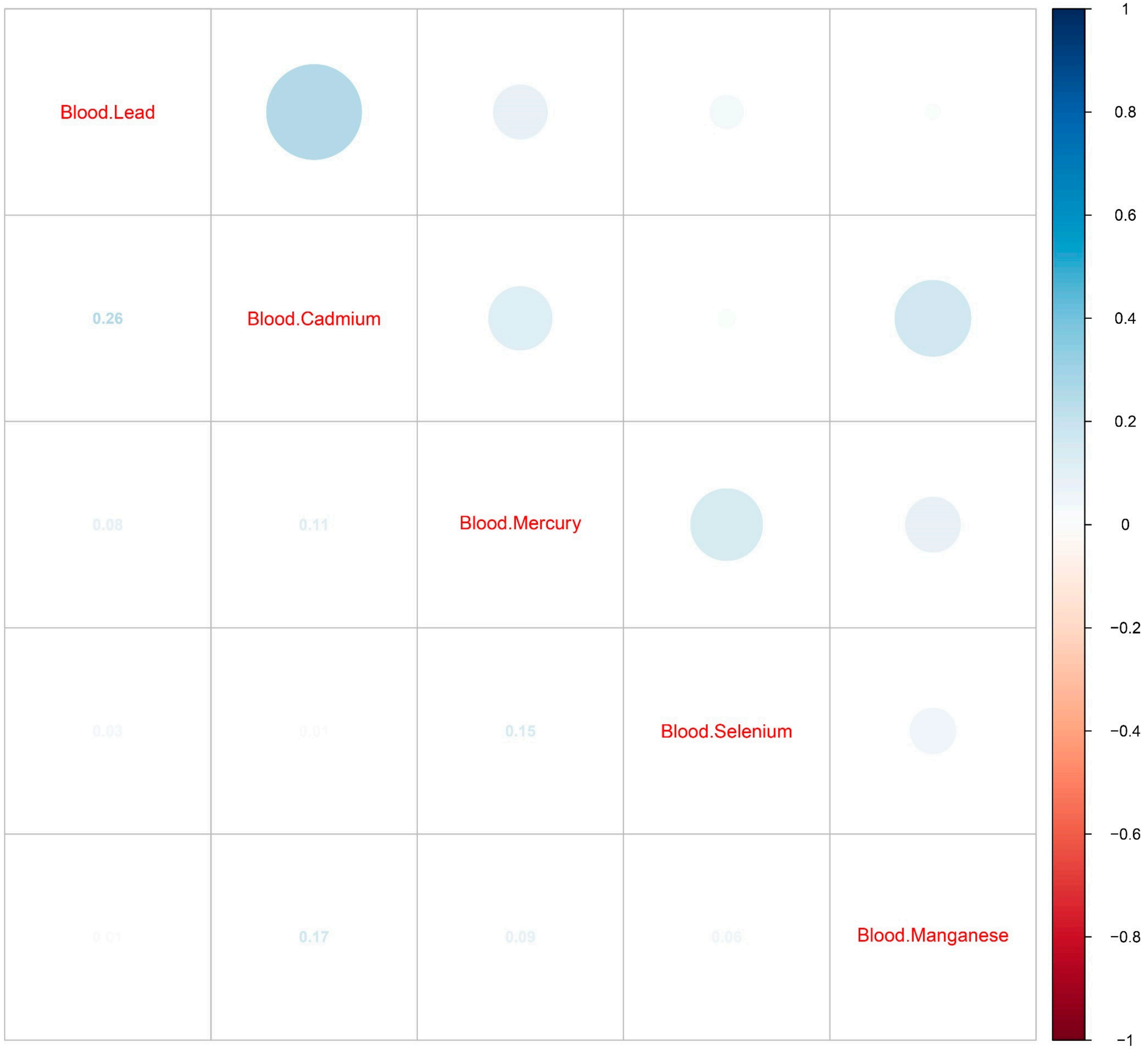

Figure S4. Combined effects of the metal as a mixture on cognitive function in males.

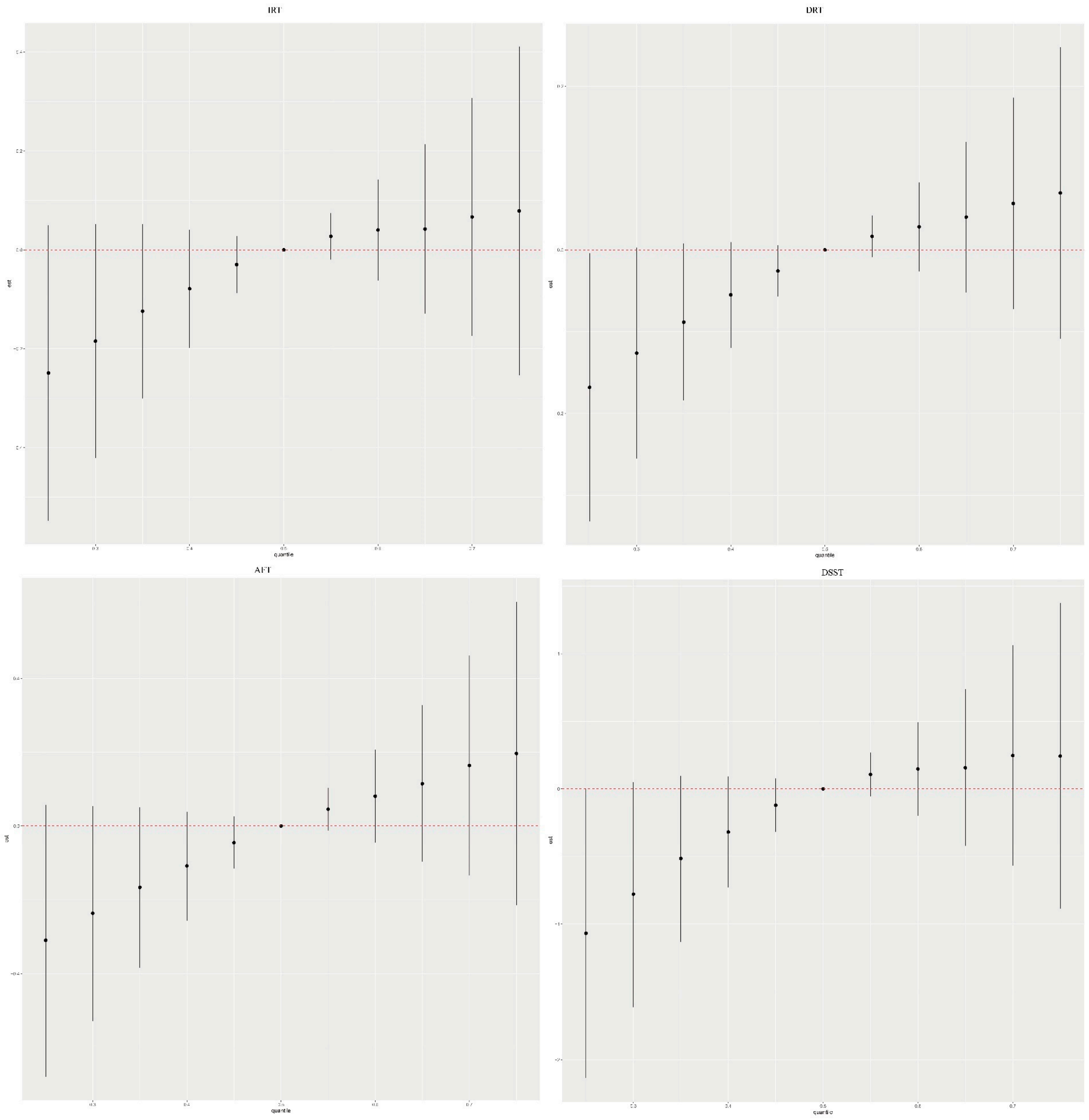

Figure S5. Combined effects of the metal as a mixture on cognitive function in females.

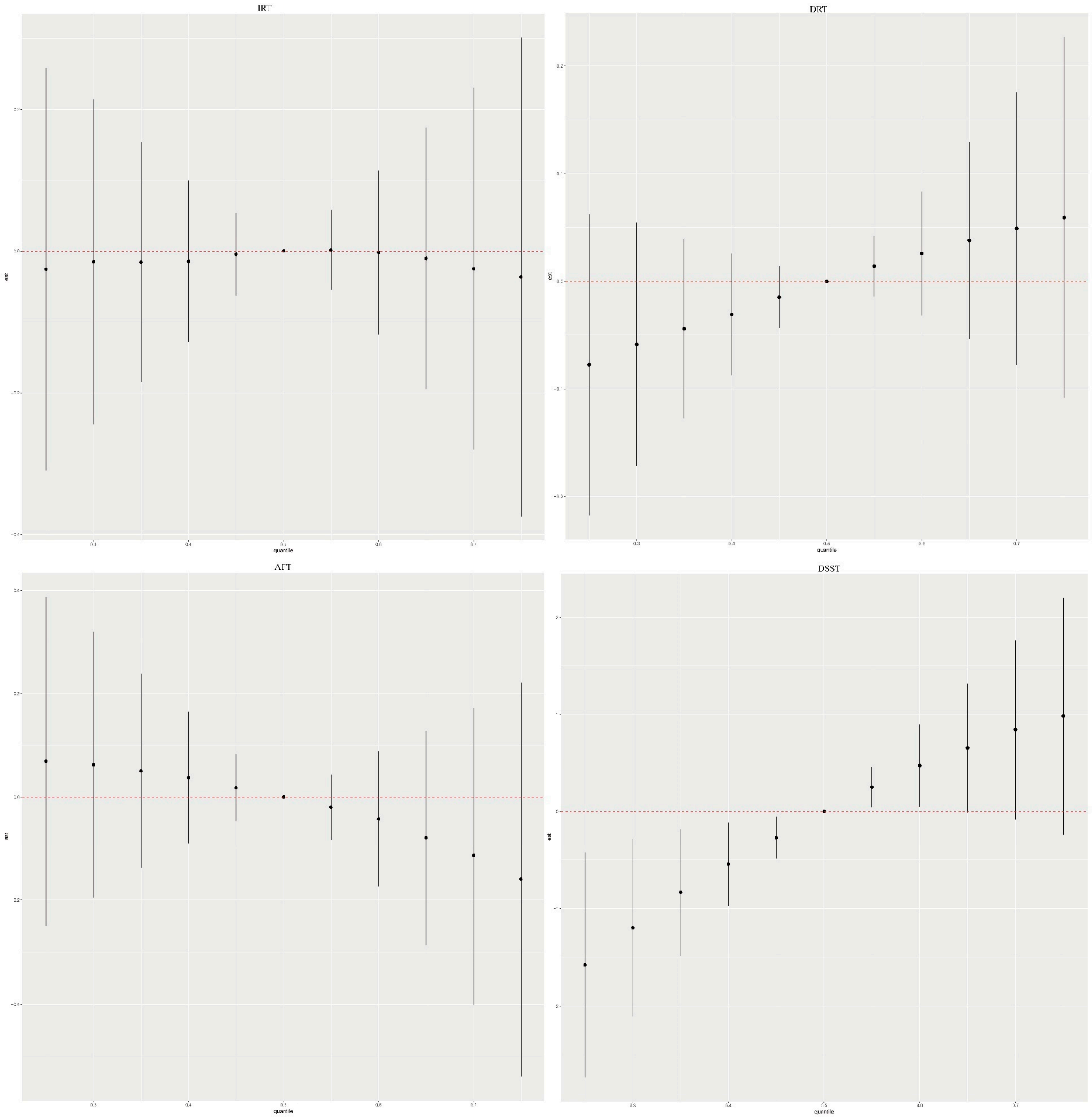

Figure S6. Univariate exposure-response functions and 95% confidence interval for each heavy metal with the other metals fixed at the median in males. Models adjusted for age, race/ethnicity, education level, alcohol intake, smoking status.

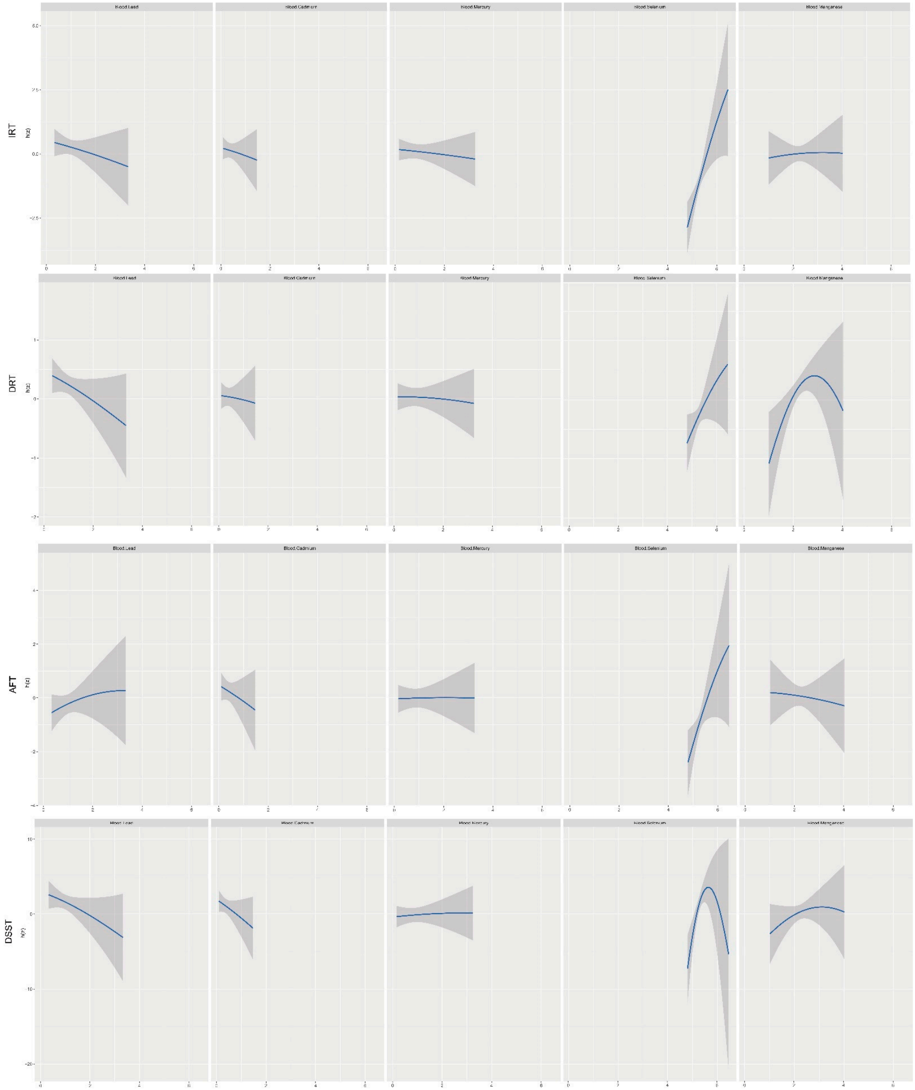

Figure S7. Univariate exposure-response functions and 95% confidence interval for each heavy metal with the other metals fixed at the median in females. Models adjusted for, age, race/ethnicity, education level, alcohol intake, smoking status.

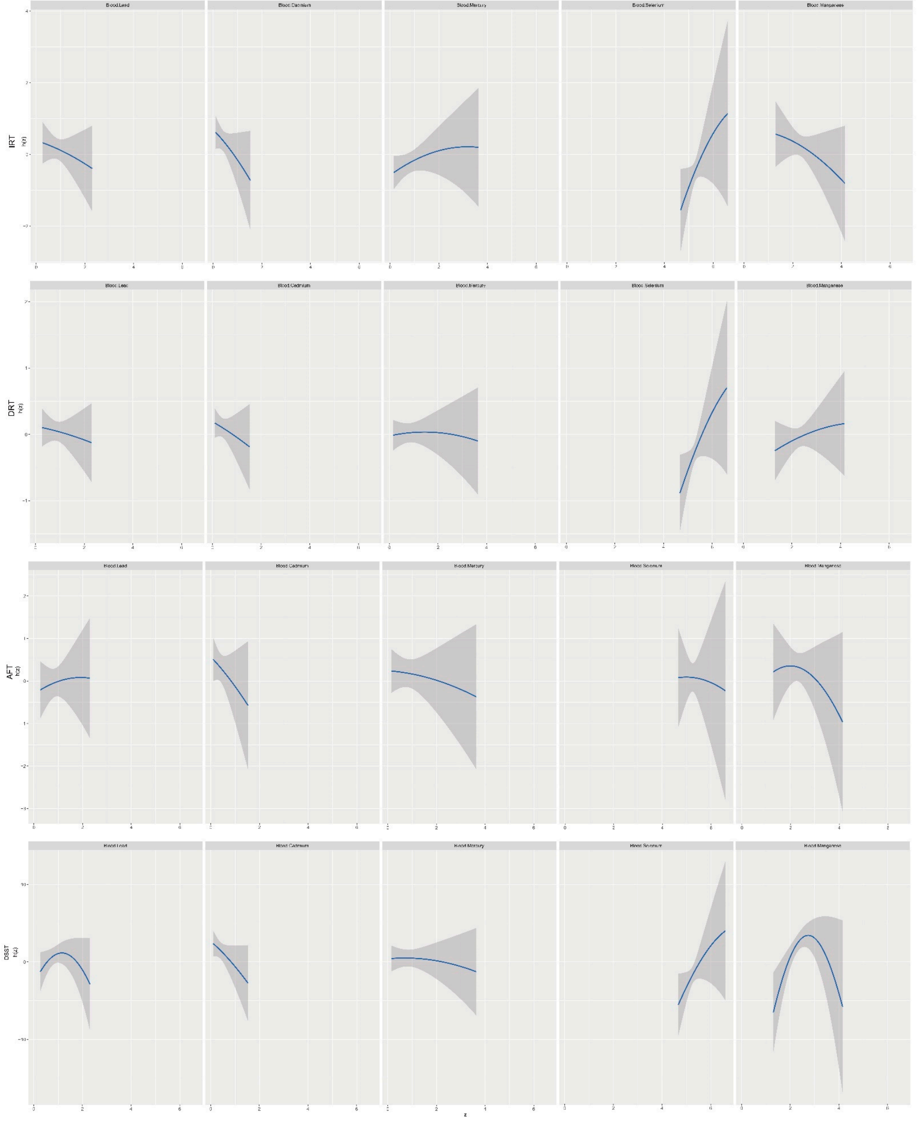

Figure S8. Estimated effects (95% CIs) of single aldehyde on cognitive function of elderly people when the levels of other aldehydes were fixing at 25th, 50th, and 75th. Models adjusted for sex, age, race/ethnicity, education level, alcohol intake, smoking status. CI, confidence interval.

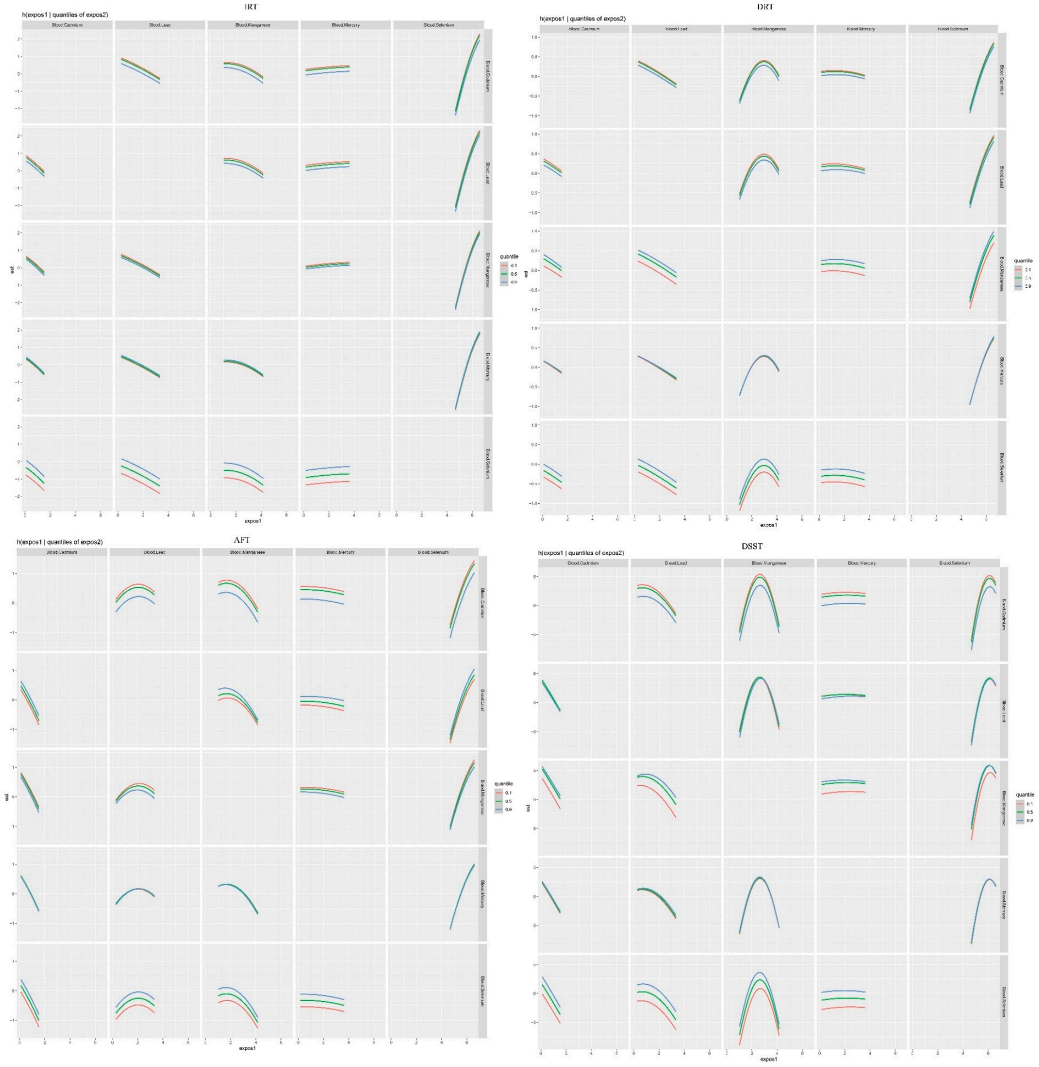

Figure S9. Estimated effects (95% CIs) of single aldehyde on cognitive function of males when the levels of other aldehydes were fixing at 25th, 50th, and 75th. Models adjusted for age, race/ethnicity, education level, alcohol intake, smoking status. CI, confidence interval.

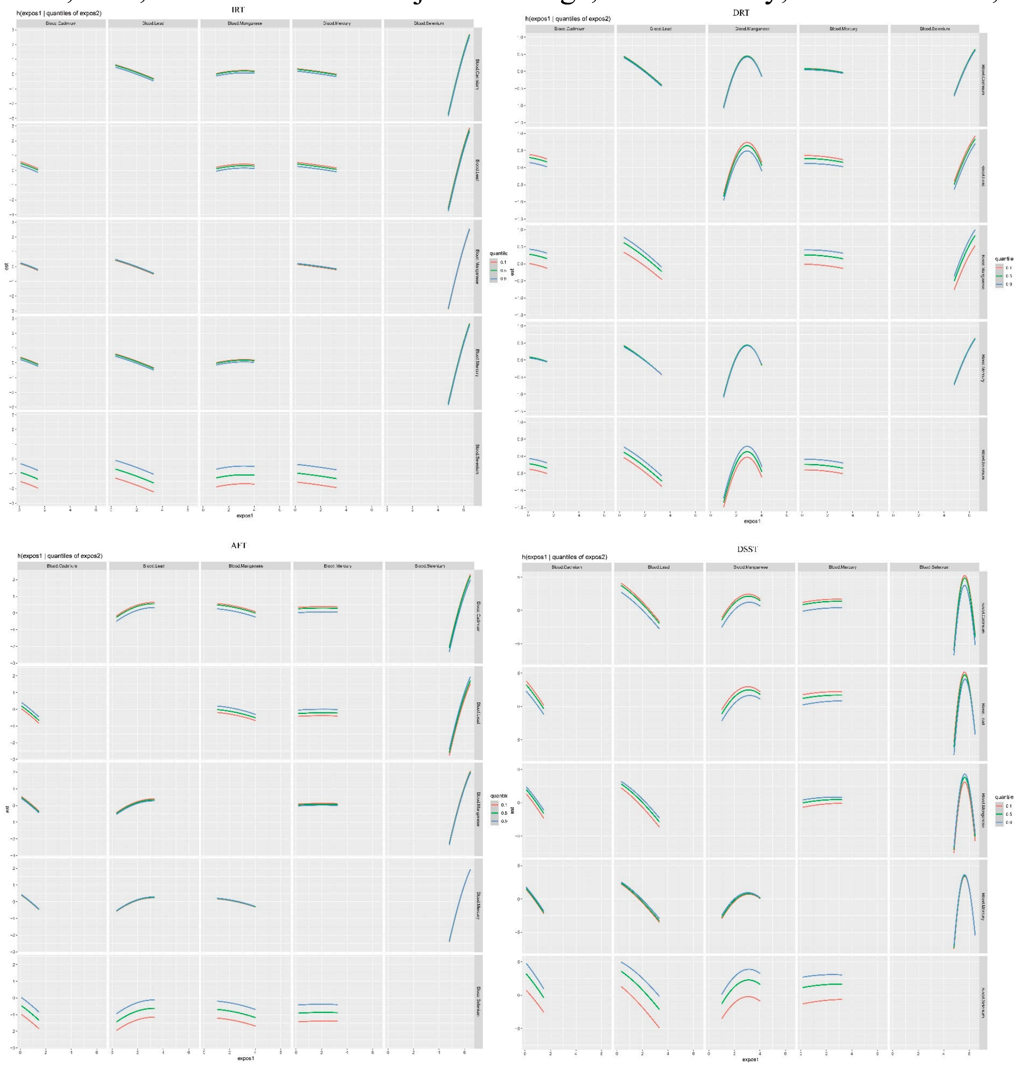

Figure S10. Estimated effects (95% CIs) of single aldehyde on cognitive function of females when the levels of other aldehydes were fixing at 25th, 50th, and 75th. Models adjusted for age, race/ethnicity, education level, alcohol intake, smoking status. CI, confidence interval.

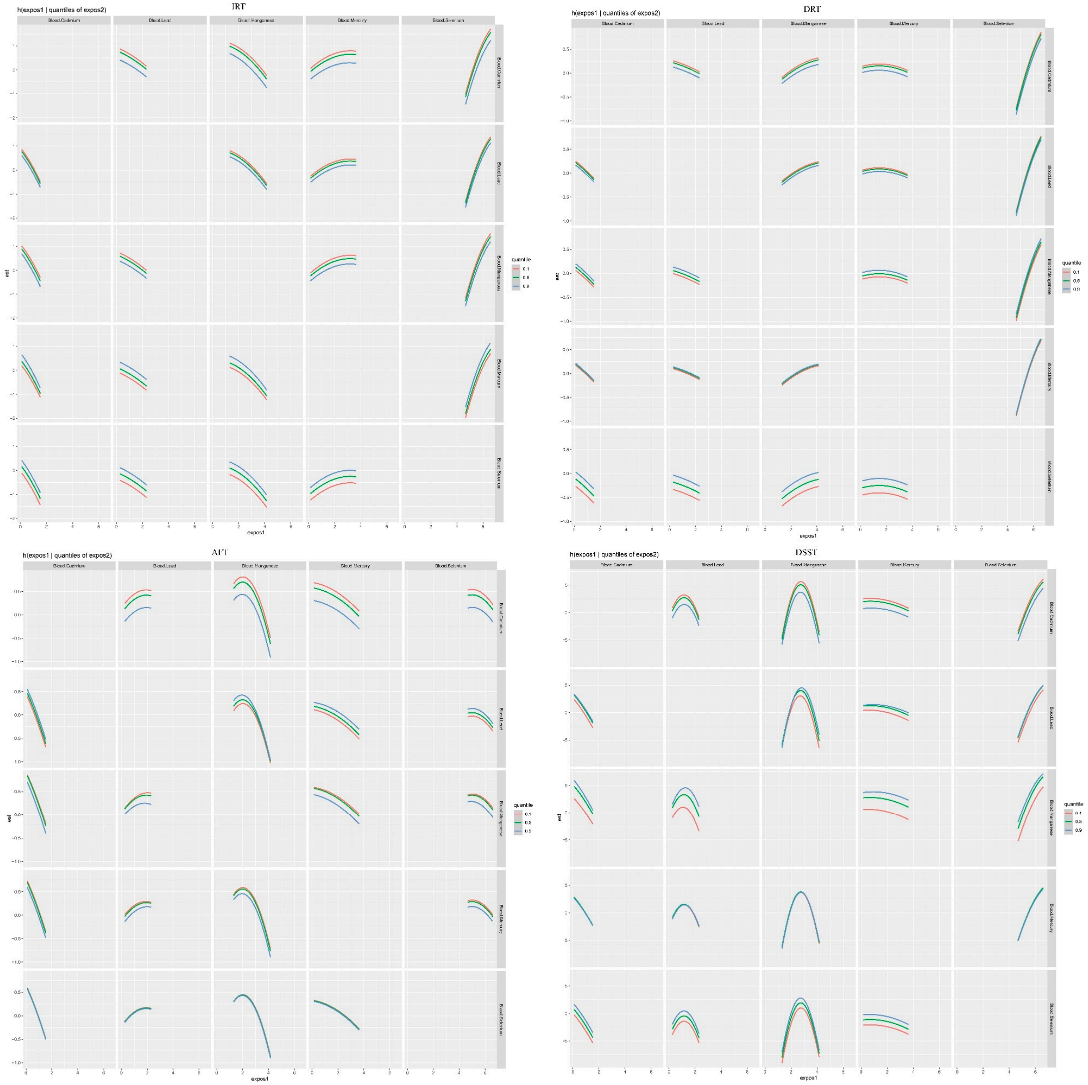

Figure S11. WQS model regression index weights for total four cognitive tests in males. The WQS models were adjusted by age, race/ethnicity, education level, alcohol intake, smoking status.

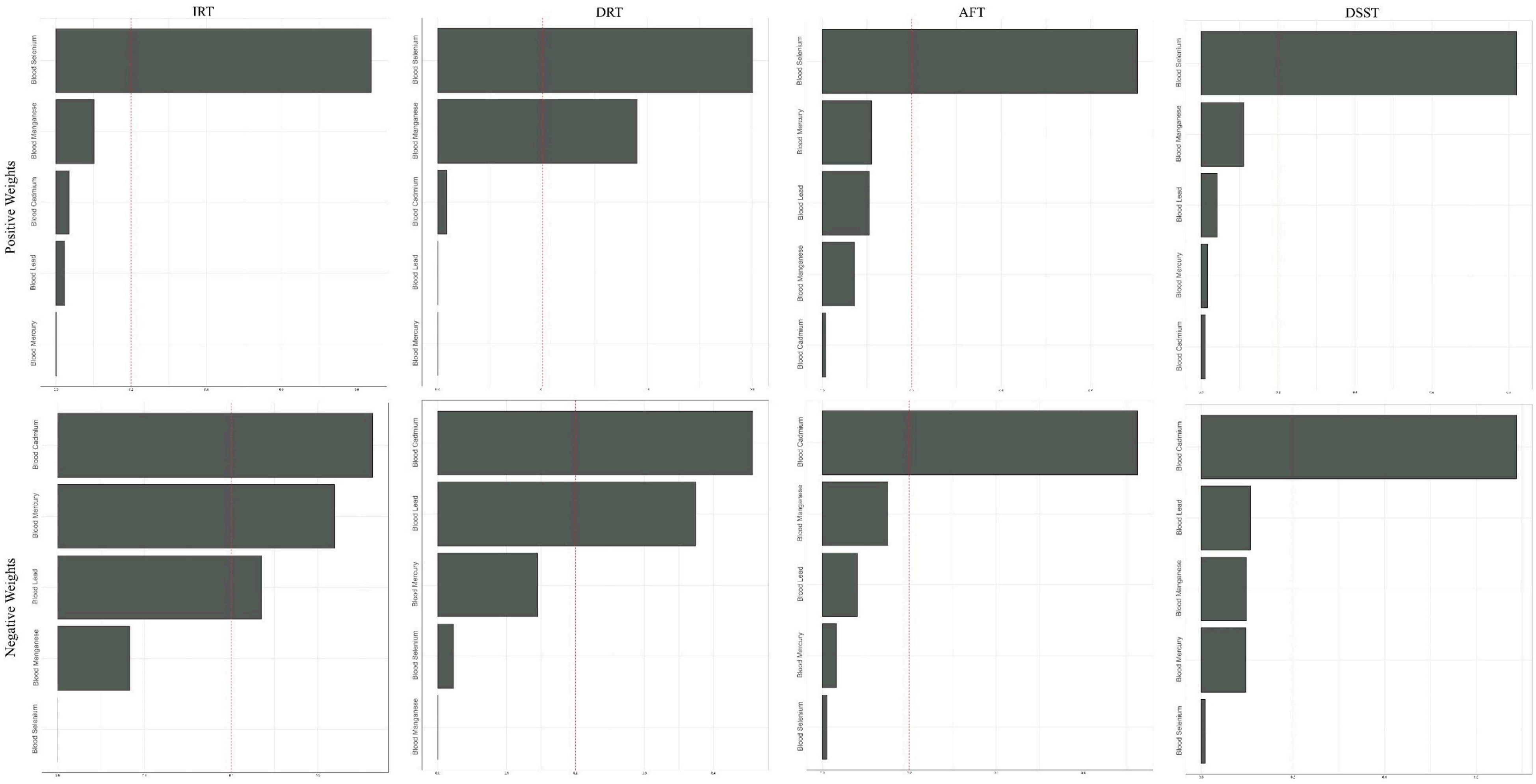

Figure S12. WQS model regression index weights for total four cognitive tests in females. The WQS models were adjusted by age, race/ethnicity, education level, alcohol intake, smoking status.

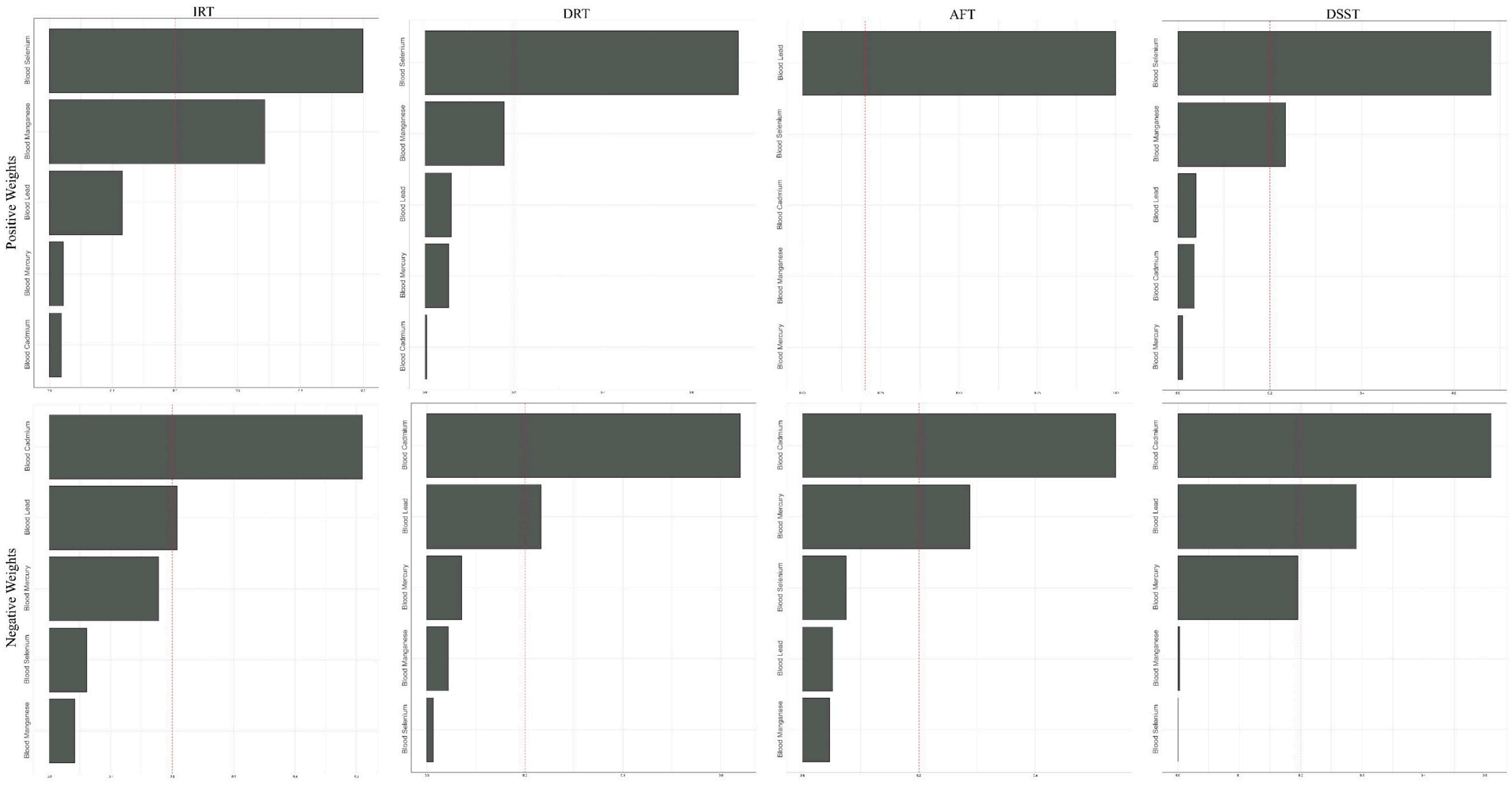

Figure S13. Qgcomp model regression joint effect (95% CI) for blood heavy metals and four cognitive tests in older people. The Qgcomp models were adjusted by sex, age, race/ethnicity, education level, alcohol intake, smoking status.

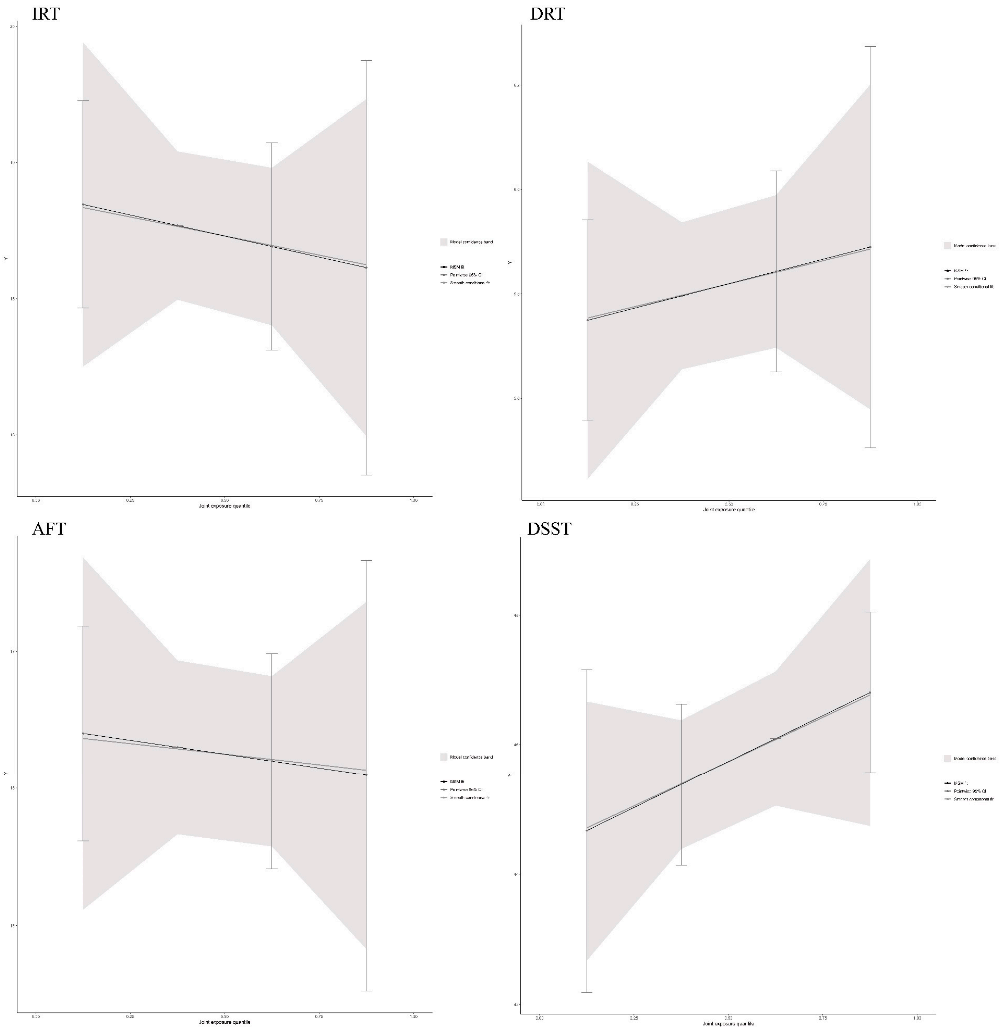

Figure S14. Qgcomp model regression joint effect (95% CI) for blood heavy metals and four cognitive tests in males. The Qgcomp models were adjusted by, age, race/ethnicity, education level, alcohol intake, smoking status.

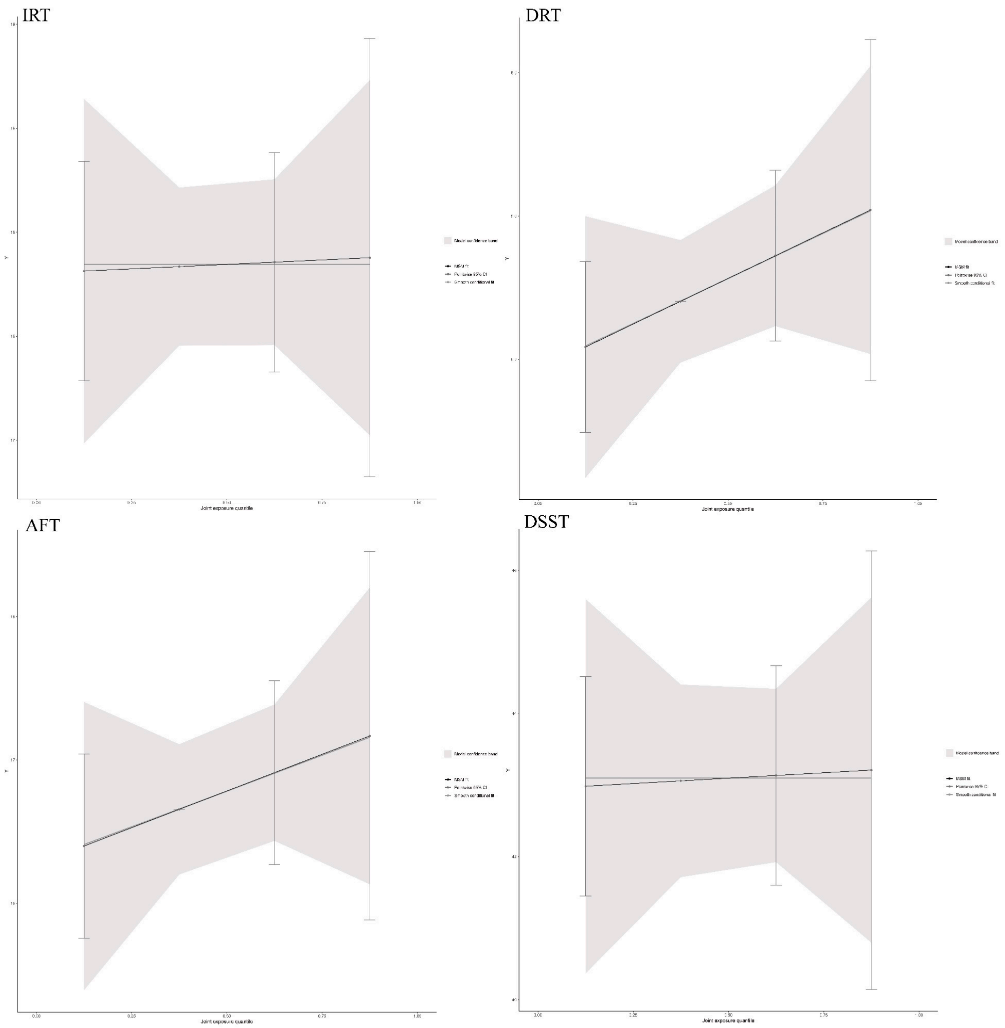

Figure S15. Qgcomp model regression joint effect (95% CI) for blood heavy metals and four cognitive tests in females. The Qgcomp models were adjusted by, age, race/ethnicity, education level, alcohol intake, smoking status.

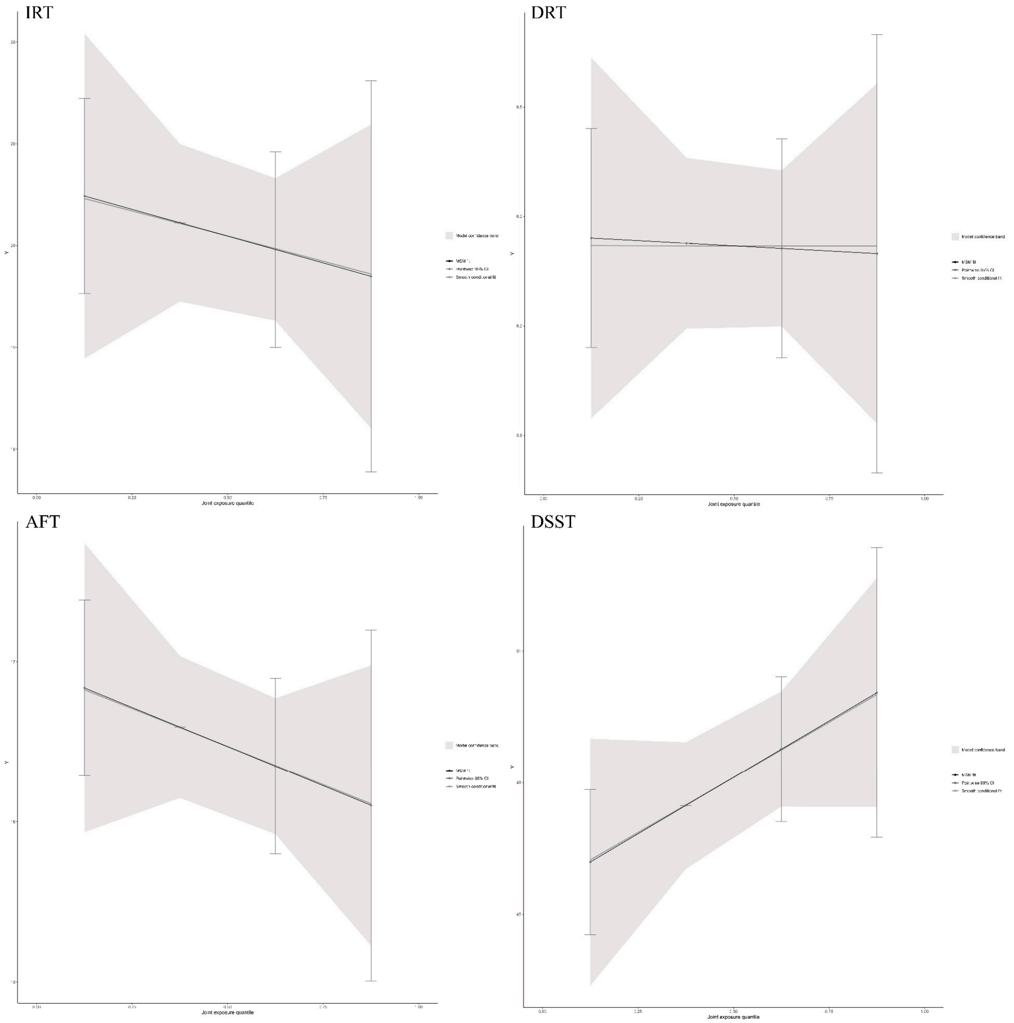

Figure S16. Qgcomp model regression index weights for blood heavy metals and four cognitive tests in males. The Qgcomp models were adjusted by age, race/ethnicity, education level, alcohol intake, smoking status.

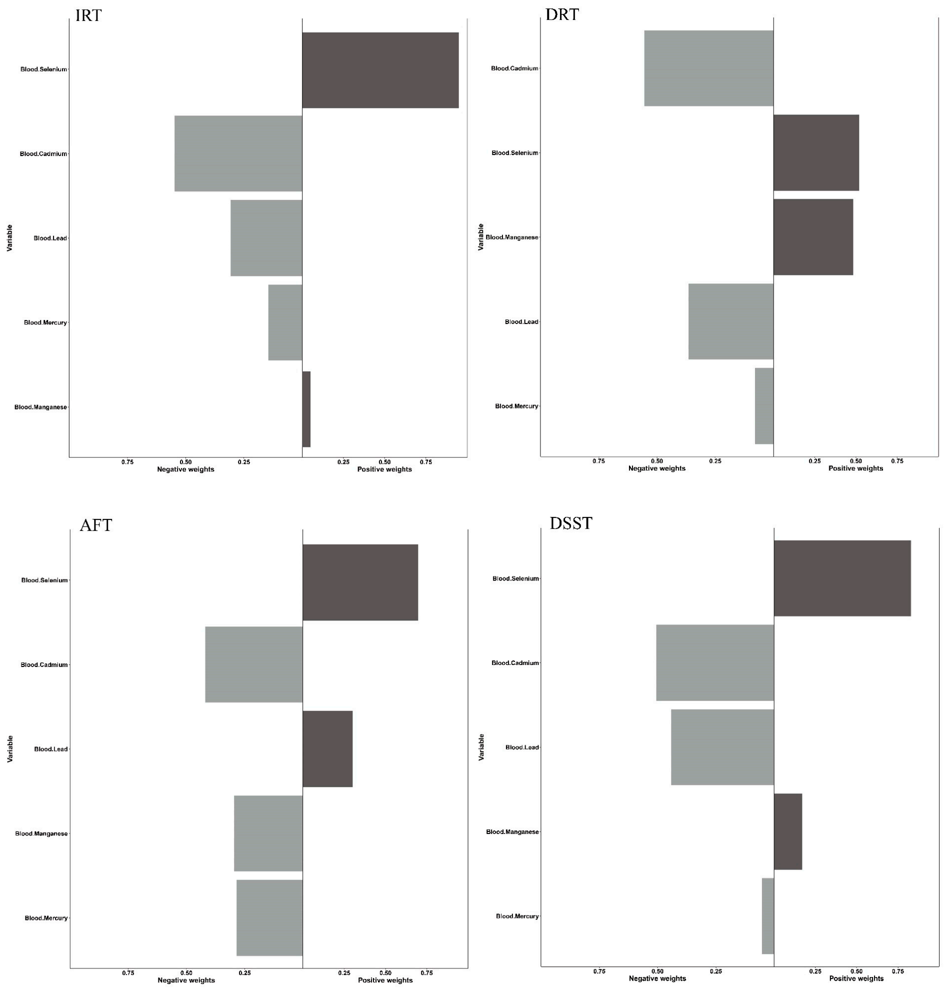

Figure S17. Qgcomp model regression index weights for blood heavy metals and four cognitive tests in females. The Qgcomp models were adjusted by age, race/ethnicity, education level, alcohol intake, smoking status.

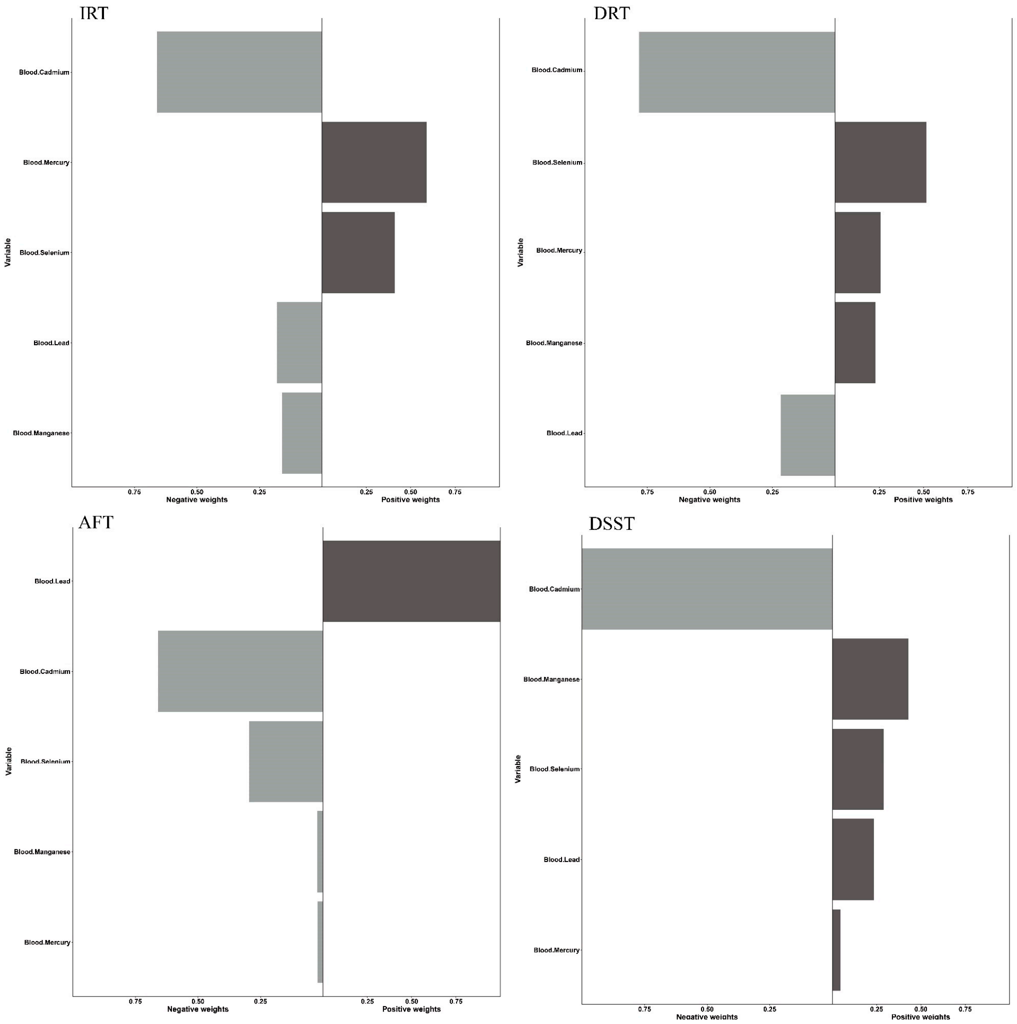

Supplement: Supplementary file 1 [file nutrients-15-02874-s001.zip › nutrients-2427872-supplementary.pdf]
